# Supplementary material for: SMartini: automated small molecule parametrization for Martini 3 force field
Source: bioRxiv. 2026 Oct 1:2026.09.29.755481. Preprint. [Version 1] doi: 10.64898/2026.09.29.755481 (PMC13648609; doi:10.64898/2026.09.29.755481)
Supplement: Supplementary file 1 [file media-1.pdf]

Supporting Information: Automated Small Molecule Parametrization for Martini 3 Force Field

S1. Internal Wasserstein Distance

The agreement between all-atom (AA) and coarse-grained (CG) conformational distributions for each bonded term was quantified using the 1-Wasserstein (earth-mover's) distance. For two one-dimensional distributions with cumulative distribution functions $F(x)$ and $G(x)$, the 1-Wasserstein distance is defined as

$$W_1(F, G) = \int_{-\infty}^{\infty} |F(x) - G(x)| \, dx. \tag{1}$$

This metric has a natural geometric interpretation: it measures the minimal "cost" of transporting the probability mass of one distribution into the shape of the other, where the cost is proportional to the distance the mass must be moved. For bonds and constraints, the Wasserstein distance was computed directly on the distance distributions (in nm). For angles, the distributions over $[0, \pi]$ were compared, and for dihedrals, the circular nature of the distributions over $[-\pi, \pi]$ was respected by evaluating the distance on the periodically wrapped cumulative distributions.

To facilitate comparison across terms with different physical units and scales, each Wasserstein distance was normalized by the combined data range of the two distributions:

$$\tilde{W}_1 = \frac{W_1(F, G)}{\max(\text{range}_F, \text{range}_G)}, \tag{2}$$

where $\text{range}_F = \max(F) - \min(F)$. A normalized distance of zero indicates identical distributions; values below approximately 0.05 are considered to reflect satisfactory agreement at CG resolution.

S2. Error Estimation (Bootstrap Resampling)

All reported means are accompanied by 95% confidence intervals estimated via bootstrap resampling. For each quantity (SASA, RMSD, Wasserstein distance), the trajectory data were resampled with replacement 1 000 times, and the mean was recomputed for each bootstrap replicate. The lower and upper bounds of the 95% confidence interval were taken as the 2.5th and 97.5th percentiles of the bootstrap distribution, respectively. This non-parametric approach makes no assumptions about the underlying distribution of the data and is robust to the autocorrelation present in MD trajectories because the bootstrap operates at the level of trajectory frames, which are treated as exchangeable observations after adequate equilibration.

S3. Solvent-Accessible Surface Area

The solvent-accessible surface area (SASA) was computed using the Shrake–Rupley algorithm (Shrake and Rupley 1973) as implemented in MDTraj (McGibbon et al. 2015). For all-atom trajectories, a probe radius of 0.14 nm was used, corresponding to the approximate radius of a water molecule. For coarse-grained trajectories, each Martini bead was treated as a single sphere with its assigned van der Waals radius (0.17–0.235 nm depending on bead type), and the same 0.14 nm probe radius was employed.

S4. Root-Mean-Square Deviation

The root-mean-square deviation (RMSD) of CG bead positions relative to the mapped AA reference was computed as

$$\mathrm{RMSD}(t) = \sqrt{\frac{1}{N}\sum_{i=1}^{N}\left\|\mathbf{r}_i^{\mathrm{CG}}(t) - \mathbf{r}_i^{\mathrm{ref}}\right\|^2}, \tag{3}$$

where N is the number of CG beads, $\mathbf{r}_i^{\mathrm{CG}}(t)$ is the position of bead i at time t in the CG trajectory, and $\mathbf{r}_i^{\mathrm{ref}}$ is the reference position of bead i obtained by mapping the equilibrium AA structure onto the CG representation. The RMSD was computed after optimal rigid-body alignment of each CG frame to the reference structure, using only the CG bead coordinates.

S5. Intermolecular Contact Maps and Fraction of Native Contacts

To evaluate how well the CG model reproduces the protein–ligand binding interface observed in atomistic simulations, we compute intermolecular contact maps and the fraction of native contacts $Q(t)$ (Best et al. 2013). A contact is defined between a ligand bead i and a protein residue j when their distance is within a cutoff $d_c = 0.6$ nm, corresponding to the typical Martini nonbonded cutoff range. This bead-to-residue resolution is chosen to make the analysis

directly comparable across CG and AA representations: in both cases the protein is reduced to the centre-of-geometry (COG) of each residue, and the ligand coordinates are expressed at the CG-bead level (using the ITP mapping to reduce AA ligand atoms to bead COGs).

For each frame t, the binary contact matrix $C_{ij}(t)$ is

$$C_{ij}(t) = \begin{cases} 1, & \text{if } \|\mathbf{r}_i^{\text{lig}}(t) - \mathbf{r}_j^{\text{prot}}(t)\| \leq d_c, \\ 0, & \text{otherwise,} \end{cases} \tag{4}$$

where $\mathbf{r}_i^{\text{lig}}$ is the position of ligand bead i and $\mathbf{r}_j^{\text{prot}}$ is the COG of protein residue j. The set of *native contacts* $\mathcal{N}$ is defined as the set of bead–residue pairs in contact in the reference (first) frame of the trajectory:

$$\mathcal{N} = \{(i, j) \mid C_{ij}(0) = 1\}. \tag{5}$$

The fraction of native contacts at time t is then

$$Q(t) = \frac{|\mathcal{N}(t)|}{|\mathcal{N}(0)|}, \quad \text{where } \mathcal{N}(t) = \{(i, j) \in \mathcal{N} \mid C_{ij}(t) = 1\}. \tag{6}$$

$Q(t)$ ranges from 0 (complete loss of the native interface) to 1 (perfect preservation) and provides a simple, interpretable measure of binding-site stability over the course of the simulation.

To focus the analysis on the binding pocket, we apply a proximity filter: only protein residues whose COG lies within 1.0 nm of any ligand bead in the reference frame are retained. This spatial pre-filtering removes residues that are far from the binding site and could otherwise dominate the contact statistics. To ensure that CG and AA contact maps are directly comparable, the residue selection is further unified: only residues that pass the 1.0 nm proximity filter in *both* representations are included in the final contact matrices.

Trajectory I/O and atom selection for CG systems were handled using MDAnalysis (Gowers et al. 2016); all-atom trajectory processing used MDTraj (McGibbon et al. 2015). Per-frame pairwise distance matrices were computed in a vectorised form using NumPy (Harris et al. 2020). Contact frequency maps were visualised as heatmaps with identical axes for CG and AA, enabling a side-by-side comparison of the binding interface sampled by each representation.

S6. Supporting Figures

ANP

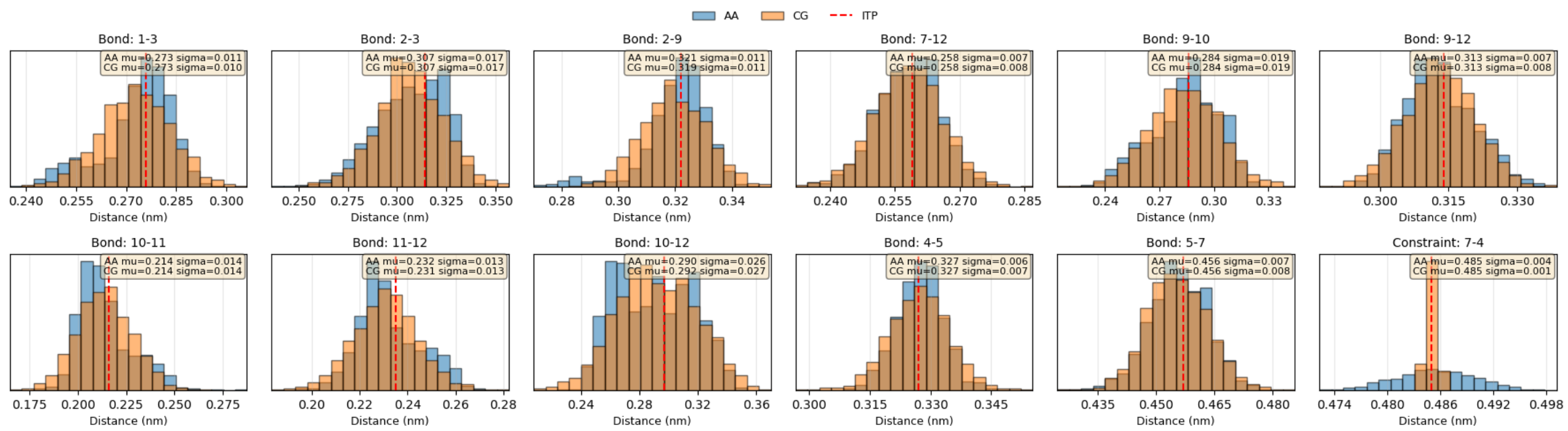

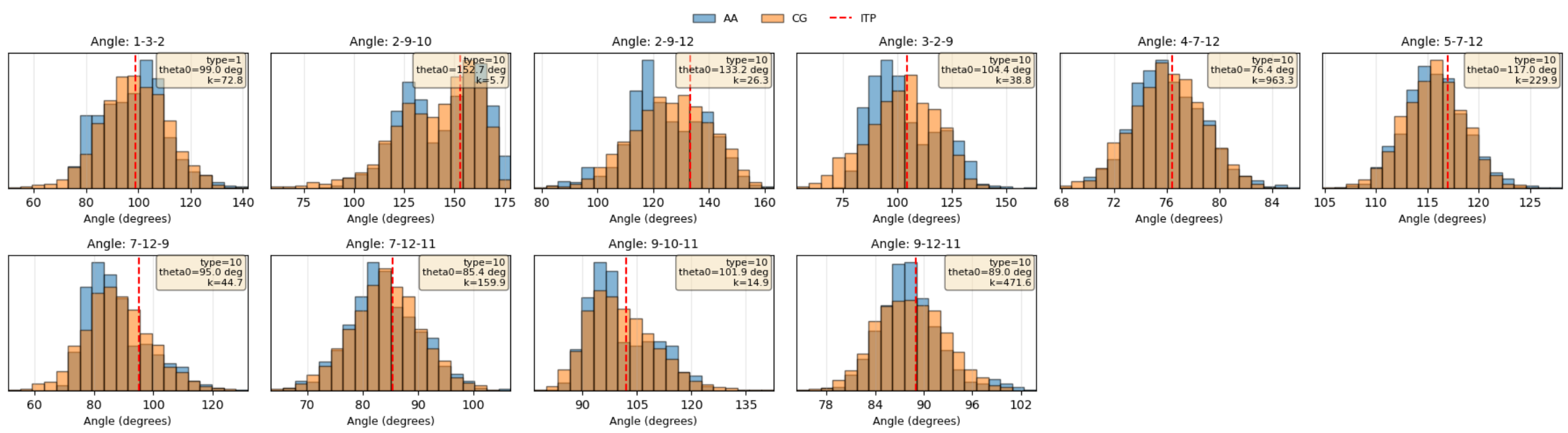

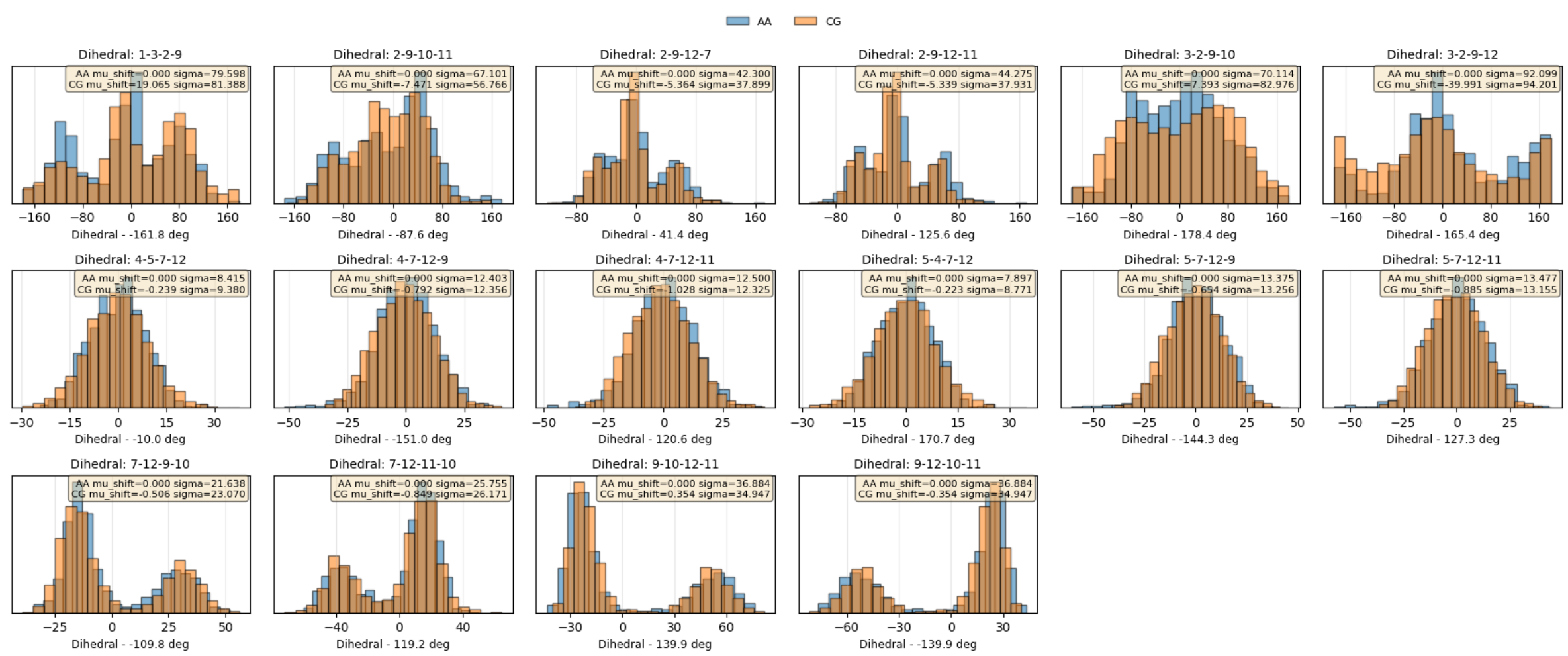

CLA

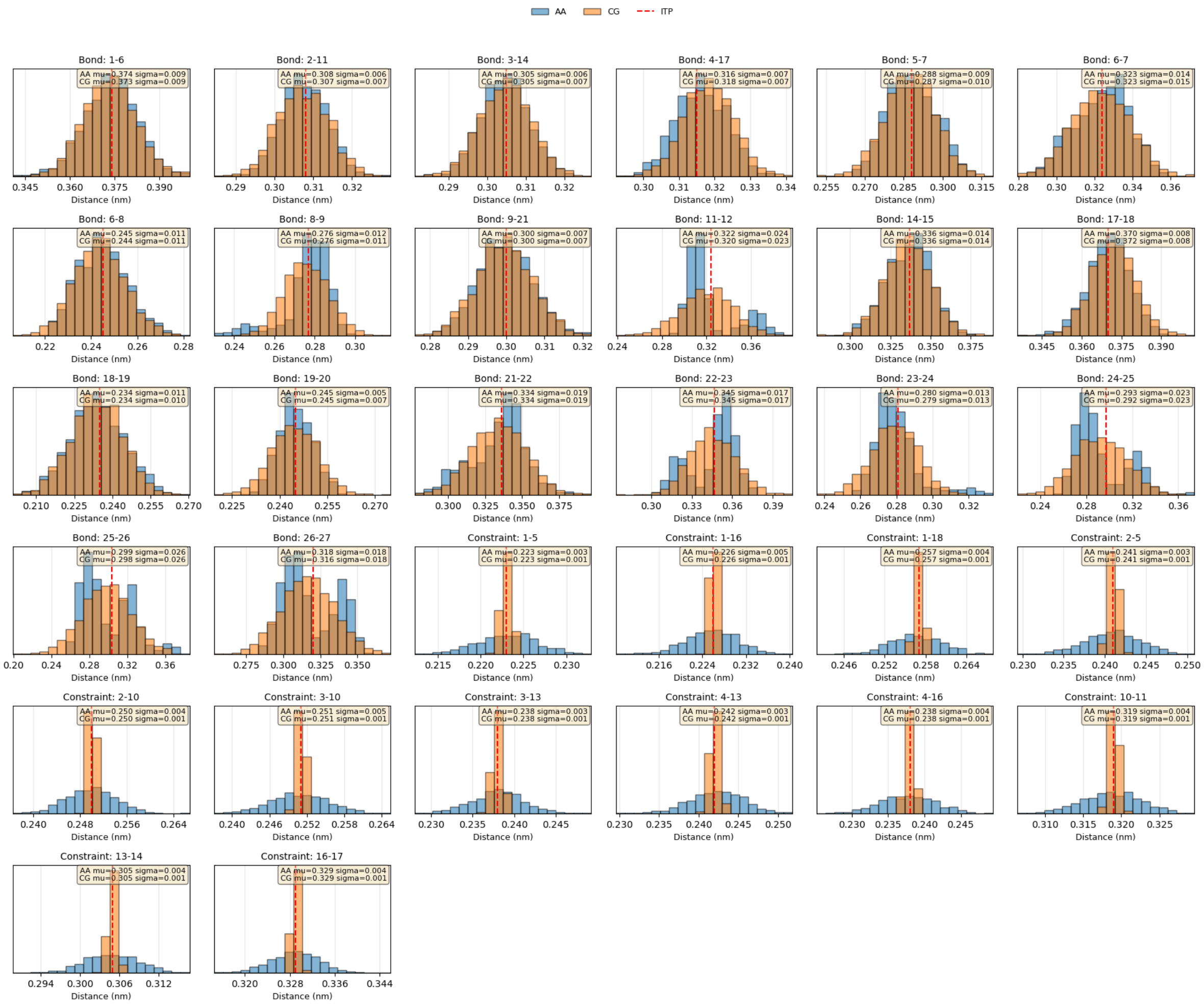
AA
CG
ITP
Bond: 1-6
AA mu=0.374 sigma=0.009
CG mu=0.373 sigma=0.009
Bond: 2-11
AA mu=0.308 sigma=0.006
CG mu=0.307 sigma=0.007
Bond: 3-14
AA mu=0.305 sigma=0.006
CG mu=0.305 sigma=0.007
Bond: 4-17
AA mu=0.316 sigma=0.007
CG mu=0.318 sigma=0.007
Bond: 5-7
AA mu=0.288 sigma=0.009
CG mu=0.287 sigma=0.010
Bond: 6-7
AA mu=0.323 sigma=0.014
CG mu=0.323 sigma=0.015
Bond: 6-8
AA mu=0.245 sigma=0.011
CG mu=0.244 sigma=0.011
Bond: 8-9
AA mu=0.276 sigma=0.012
CG mu=0.276 sigma=0.011
Bond: 9-21
AA mu=0.300 sigma=0.007
CG mu=0.300 sigma=0.007
Bond: 11-12
AA mu=0.322 sigma=0.024
CG mu=0.320 sigma=0.023
Bond: 14-15
AA mu=0.336 sigma=0.014
CG mu=0.336 sigma=0.014
Bond: 17-18
AA mu=0.370 sigma=0.008
CG mu=0.372 sigma=0.008
Bond: 18-19
AA mu=0.234 sigma=0.011
CG mu=0.234 sigma=0.010
Bond: 19-20
AA mu=0.245 sigma=0.005
CG mu=0.245 sigma=0.007
Bond: 21-22
AA mu=0.334 sigma=0.019
CG mu=0.334 sigma=0.019
Bond: 22-23
AA mu=0.345 sigma=0.017
CG mu=0.345 sigma=0.017
Bond: 23-24
AA mu=0.280 sigma=0.013
CG mu=0.279 sigma=0.013
Bond: 24-25
AA mu=0.293 sigma=0.023
CG mu=0.292 sigma=0.023
Bond: 25-26
AA mu=0.299 sigma=0.026
CG mu=0.298 sigma=0.026
Bond: 26-27
AA mu=0.318 sigma=0.018
CG mu=0.316 sigma=0.018
Constraint: 1-5
AA mu=0.223 sigma=0.003
CG mu=0.223 sigma=0.001
Constraint: 1-16
AA mu=0.226 sigma=0.005
CG mu=0.226 sigma=0.001
Constraint: 1-18
AA mu=0.257 sigma=0.004
CG mu=0.257 sigma=0.001
Constraint: 2-5
AA mu=0.241 sigma=0.003
CG mu=0.241 sigma=0.001
Constraint: 2-10
AA mu=0.250 sigma=0.004
CG mu=0.250 sigma=0.001
Constraint: 3-10
AA mu=0.251 sigma=0.005
CG mu=0.251 sigma=0.001
Constraint: 3-13
AA mu=0.238 sigma=0.003
CG mu=0.238 sigma=0.001
Constraint: 4-13
AA mu=0.242 sigma=0.003
CG mu=0.242 sigma=0.001
Constraint: 4-16
AA mu=0.238 sigma=0.004
CG mu=0.238 sigma=0.001
Constraint: 10-11
AA mu=0.319 sigma=0.004
CG mu=0.319 sigma=0.001
Constraint: 13-14
AA mu=0.305 sigma=0.004
CG mu=0.305 sigma=0.001
Constraint: 16-17
AA mu=0.329 sigma=0.004
CG mu=0.329 sigma=0.001
Distance (nm)

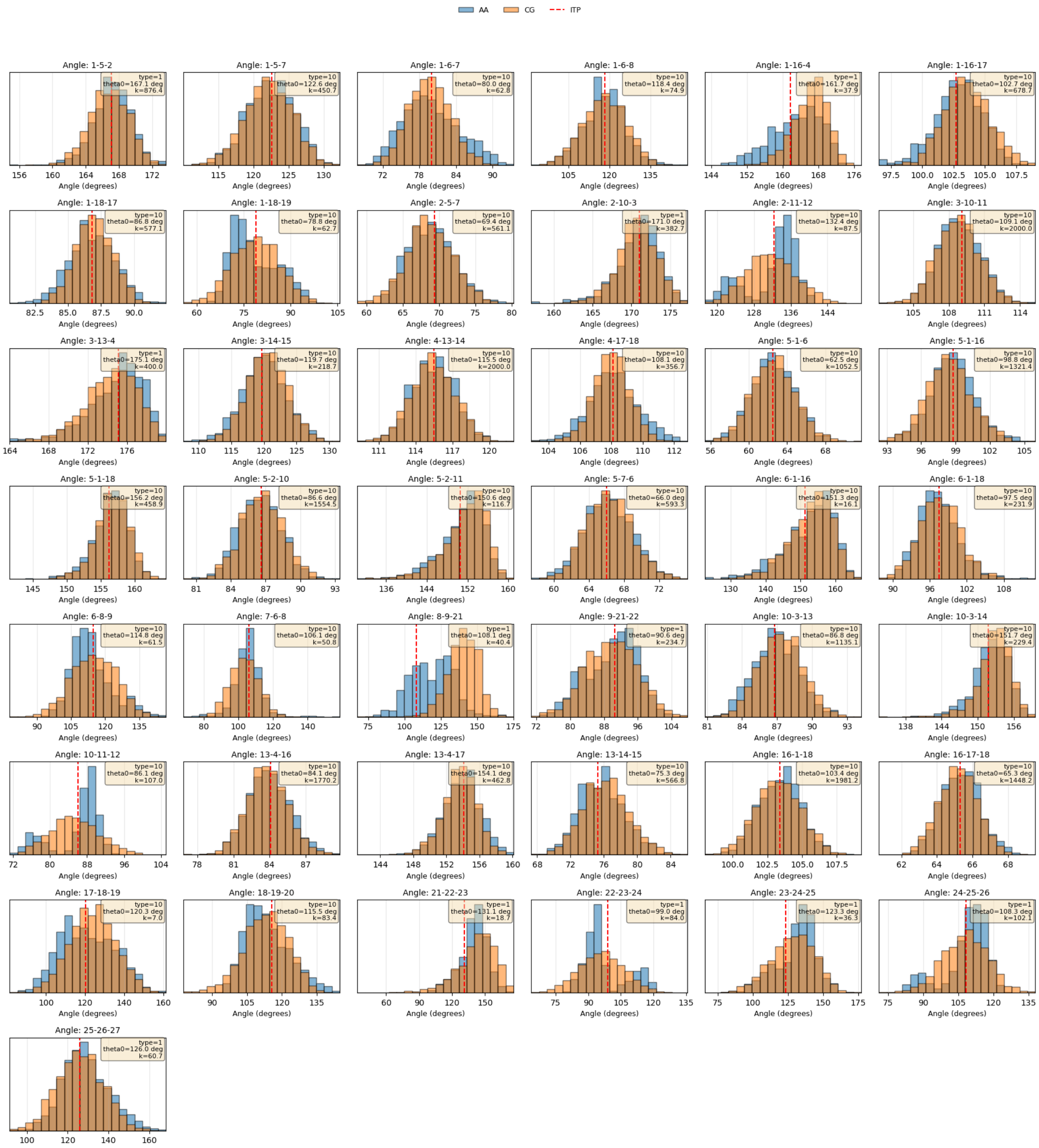
AA
CG
ITP
Angle: 1-5-2
type=1 theta0=167.1 deg k=876.4
Angle: 1-5-7
type=10 theta0=122.6 deg k=450.7
Angle: 1-6-7
type=10 theta0=80.0 deg k=62.8
Angle: 1-6-8
type=10 theta0=118.4 deg k=74.9
Angle: 1-16-4
type=1 theta0=161.7 deg k=37.9
Angle: 1-16-17
type=10 theta0=102.7 deg k=678.7
Angle: 1-18-17
type=10 theta0=86.8 deg k=577.1
Angle: 1-18-19
type=10 theta0=78.8 deg k=62.7
Angle: 2-5-7
type=10 theta0=69.4 deg k=561.1
Angle: 2-10-3
type=1 theta0=171.0 deg k=382.7
Angle: 2-11-12
type=10 theta0=132.4 deg k=87.5
Angle: 3-10-11
type=10 theta0=109.1 deg k=2000.0
Angle: 3-13-4
type=1 theta0=175.1 deg k=400.0
Angle: 3-14-15
type=10 theta0=119.7 deg k=218.7
Angle: 4-13-14
type=10 theta0=115.5 deg k=2000.0
Angle: 4-17-18
type=10 theta0=108.1 deg k=356.7
Angle: 5-1-6
type=10 theta0=62.5 deg k=1052.5
Angle: 5-1-16
type=10 theta0=98.8 deg k=1321.4
Angle: 5-1-18
type=10 theta0=156.2 deg k=458.9
Angle: 5-2-10
type=10 theta0=86.6 deg k=1554.5
Angle: 5-2-11
type=10 theta0=150.6 deg k=116.7
Angle: 5-7-6
type=10 theta0=66.0 deg k=593.3
Angle: 6-1-16
type=10 theta0=151.3 deg k=16.1
Angle: 6-1-18
type=10 theta0=97.5 deg k=231.9
Angle: 6-8-9
type=10 theta0=114.8 deg k=61.5
Angle: 7-6-8
type=10 theta0=106.1 deg k=50.8
Angle: 8-9-21
type=1 theta0=108.1 deg k=40.4
Angle: 9-21-22
type=1 theta0=90.6 deg k=234.7
Angle: 10-3-13
type=10 theta0=86.8 deg k=1135.1
Angle: 10-3-14
type=10 theta0=151.7 deg k=229.4
Angle: 10-11-12
type=10 theta0=86.1 deg k=107.0
Angle: 13-4-16
type=10 theta0=84.1 deg k=1770.2
Angle: 13-4-17
type=10 theta0=154.1 deg k=462.8
Angle: 13-14-15
type=10 theta0=75.3 deg k=566.8
Angle: 16-1-18
type=10 theta0=103.4 deg k=1981.2
Angle: 16-17-18
type=10 theta0=65.3 deg k=1448.2
Angle: 17-18-19
type=10 theta0=120.3 deg k=7.0
Angle: 18-19-20
type=10 theta0=115.5 deg k=83.4
Angle: 21-22-23
type=1 theta0=131.1 deg k=18.7
Angle: 22-23-24
type=1 theta0=99.0 deg k=84.0
Angle: 23-24-25
type=1 theta0=123.3 deg k=36.3
Angle: 24-25-26
type=1 theta0=108.3 deg k=102.1
Angle: 25-26-27
type=1 theta0=126.0 deg k=60.7
Angle (degrees)

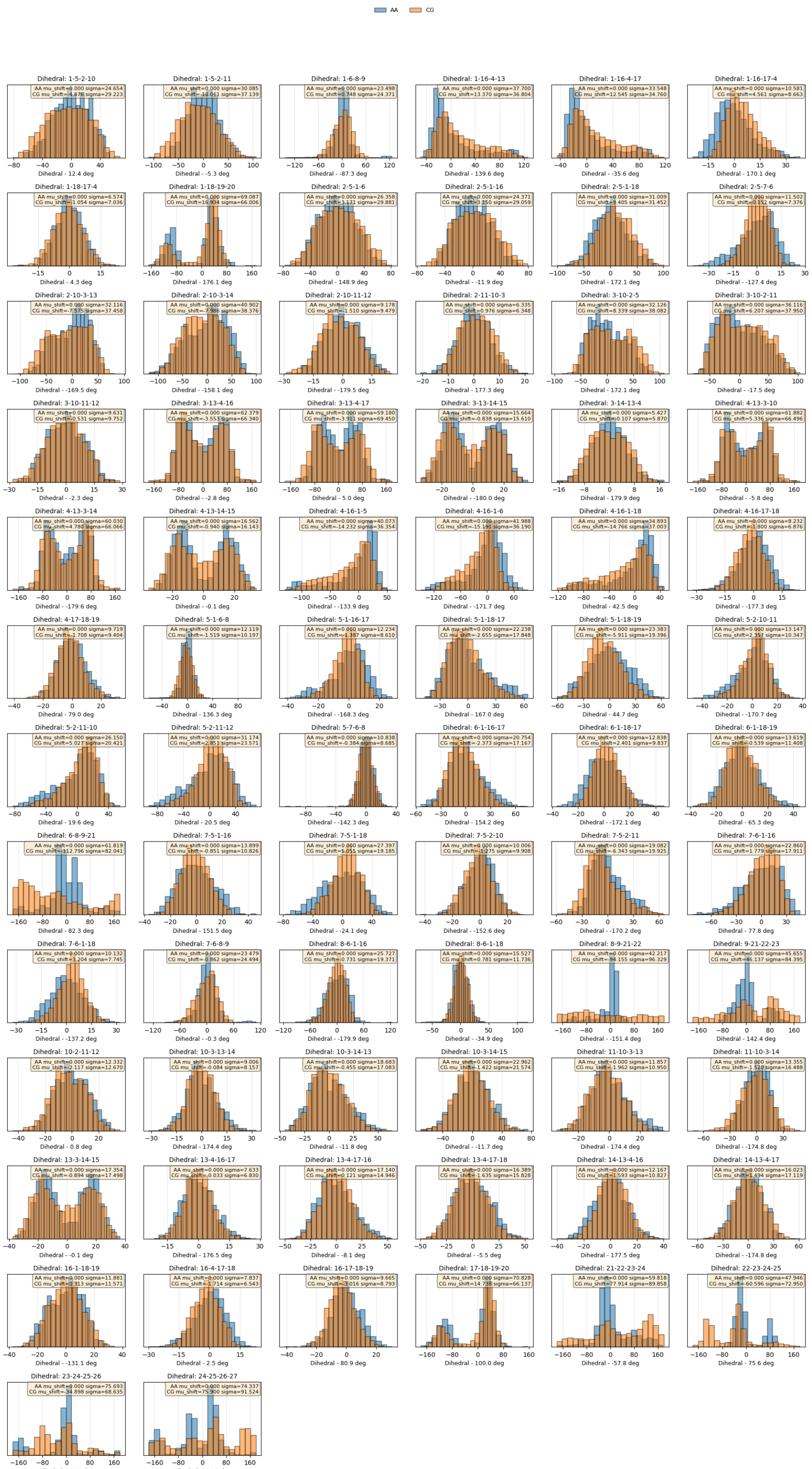
AA
CG
Dihedral: 1-5-2-10
Dihedral: 1-5-2-11
Dihedral: 1-6-8-9
Dihedral: 1-16-4-13
Dihedral: 1-16-4-17
Dihedral: 1-16-17-4
Dihedral: 1-18-17-4
Dihedral: 1-18-19-20
Dihedral: 2-5-1-6
Dihedral: 2-5-1-16
Dihedral: 2-5-1-18
Dihedral: 2-5-7-6
Dihedral: 2-10-3-13
Dihedral: 2-10-3-14
Dihedral: 2-10-11-12
Dihedral: 2-11-10-3
Dihedral: 3-10-2-5
Dihedral: 3-10-2-11
Dihedral: 3-10-11-12
Dihedral: 3-13-4-16
Dihedral: 3-13-4-17
Dihedral: 3-13-14-15
Dihedral: 3-14-13-4
Dihedral: 4-13-3-10
Dihedral: 4-13-3-14
Dihedral: 4-13-14-15
Dihedral: 4-16-1-5
Dihedral: 4-16-1-6
Dihedral: 4-16-1-18
Dihedral: 4-16-17-18
Dihedral: 4-17-18-19
Dihedral: 5-1-6-8
Dihedral: 5-1-16-17
Dihedral: 5-1-18-17
Dihedral: 5-1-18-19
Dihedral: 5-2-10-11
Dihedral: 5-2-11-10
Dihedral: 5-2-11-12
Dihedral: 5-7-6-8
Dihedral: 6-1-16-17
Dihedral: 6-1-18-17
Dihedral: 6-1-18-19
Dihedral: 6-8-9-21
Dihedral: 7-5-1-16
Dihedral: 7-5-1-18
Dihedral: 7-5-2-10
Dihedral: 7-5-2-11
Dihedral: 7-6-1-16
Dihedral: 7-6-1-18
Dihedral: 7-6-8-9
Dihedral: 8-6-1-16
Dihedral: 8-6-1-18
Dihedral: 8-9-21-22
Dihedral: 9-21-22-23
Dihedral: 10-2-11-12
Dihedral: 10-3-13-14
Dihedral: 10-3-14-13
Dihedral: 10-3-14-15
Dihedral: 11-10-3-13
Dihedral: 11-10-3-14
Dihedral: 13-3-14-15
Dihedral: 13-4-16-17
Dihedral: 13-4-17-16
Dihedral: 13-4-17-18
Dihedral: 14-13-4-16
Dihedral: 14-13-4-17
Dihedral: 16-1-18-19
Dihedral: 16-4-17-18
Dihedral: 16-17-18-19
Dihedral: 17-18-19-20
Dihedral: 21-22-23-24
Dihedral: 22-23-24-25
Dihedral: 23-24-25-26
Dihedral: 24-25-26-27

DMBI

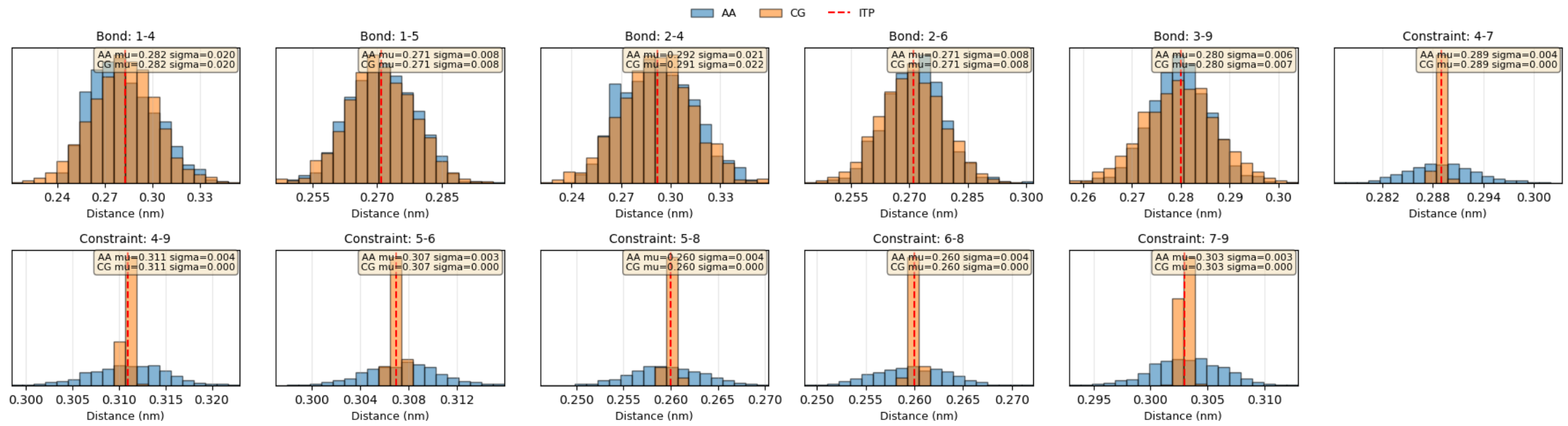
AA
CG
ITP
Bond: 1-4
Bond: 1-5
Bond: 2-4
Bond: 2-6
Bond: 3-9
Constraint: 4-7
Constraint: 4-9
Constraint: 5-6
Constraint: 5-8
Constraint: 6-8
Constraint: 7-9
Distance (nm)

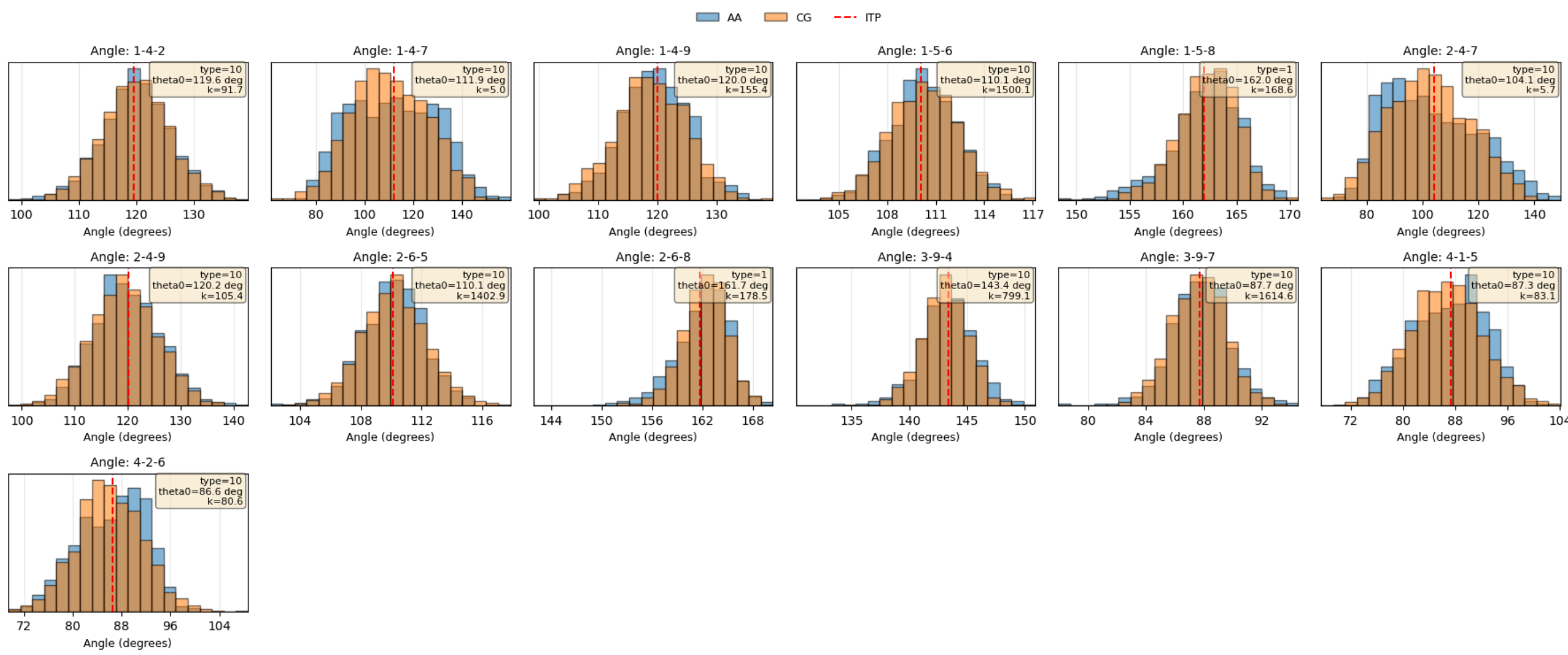
AA
CG
ITP
Angle: 1-4-2
Angle: 1-4-7
Angle: 1-4-9
Angle: 1-5-6
Angle: 1-5-8
Angle: 2-4-7
Angle: 2-4-9
Angle: 2-6-5
Angle: 2-6-8
Angle: 3-9-4
Angle: 3-9-7
Angle: 4-1-5
Angle: 4-2-6
Angle (degrees)

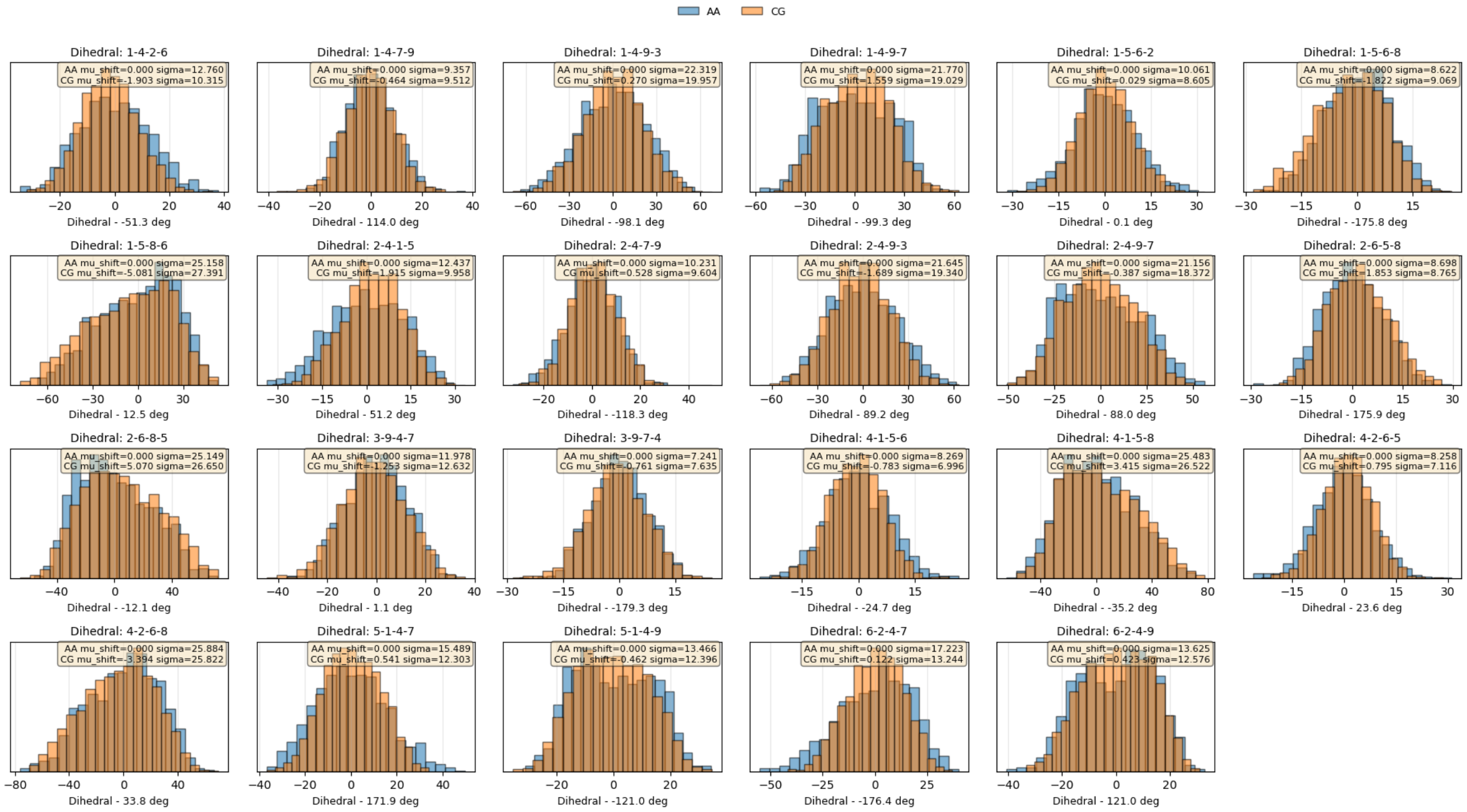
AA
CG
Dihedral: 1-4-2-6
Dihedral - -51.3 deg
Dihedral: 1-4-7-9
Dihedral - 114.0 deg
Dihedral: 1-4-9-3
Dihedral - -98.1 deg
Dihedral: 1-4-9-7
Dihedral - -99.3 deg
Dihedral: 1-5-6-2
Dihedral - 0.1 deg
Dihedral: 1-5-6-8
Dihedral - -175.8 deg
Dihedral: 1-5-8-6
Dihedral - 12.5 deg
Dihedral: 2-4-1-5
Dihedral - 51.2 deg
Dihedral: 2-4-7-9
Dihedral - -118.3 deg
Dihedral: 2-4-9-3
Dihedral - 89.2 deg
Dihedral: 2-4-9-7
Dihedral - 88.0 deg
Dihedral: 2-6-5-8
Dihedral - 175.9 deg
Dihedral: 2-6-8-5
Dihedral - -12.1 deg
Dihedral: 3-9-4-7
Dihedral - 1.1 deg
Dihedral: 3-9-7-4
Dihedral - -179.3 deg
Dihedral: 4-1-5-6
Dihedral - -24.7 deg
Dihedral: 4-1-5-8
Dihedral - -35.2 deg
Dihedral: 4-2-6-5
Dihedral - 23.6 deg
Dihedral: 4-2-6-8
Dihedral - 33.8 deg
Dihedral: 5-1-4-7
Dihedral - 171.9 deg
Dihedral: 5-1-4-9
Dihedral - -121.0 deg
Dihedral: 6-2-4-7
Dihedral - -176.4 deg
Dihedral: 6-2-4-9
Dihedral - 121.0 deg

FTA

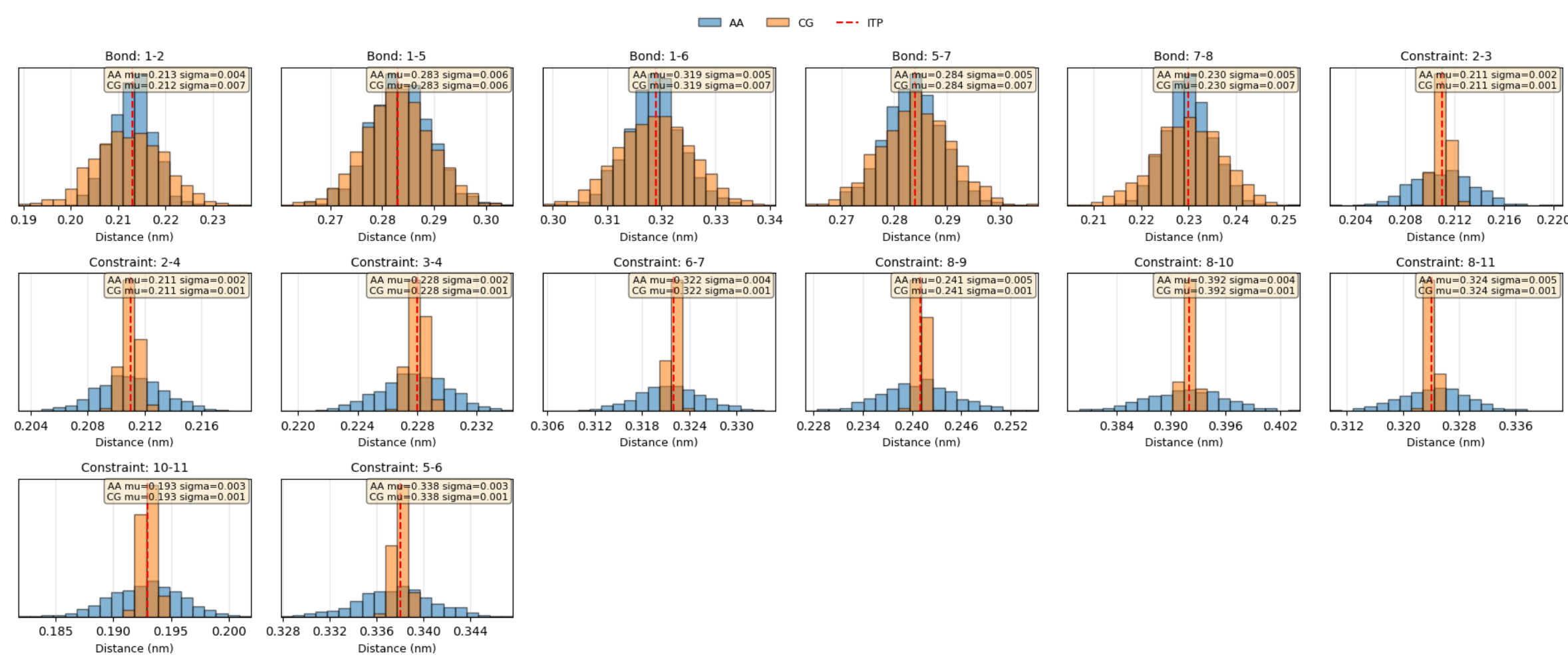
AA
CG
ITP
Bond: 1-2
Bond: 1-5
Bond: 1-6
Bond: 5-7
Bond: 7-8
Constraint: 2-3
Constraint: 2-4
Constraint: 3-4
Constraint: 6-7
Constraint: 8-9
Constraint: 8-10
Constraint: 8-11
Constraint: 10-11
Constraint: 5-6
Distance (nm)

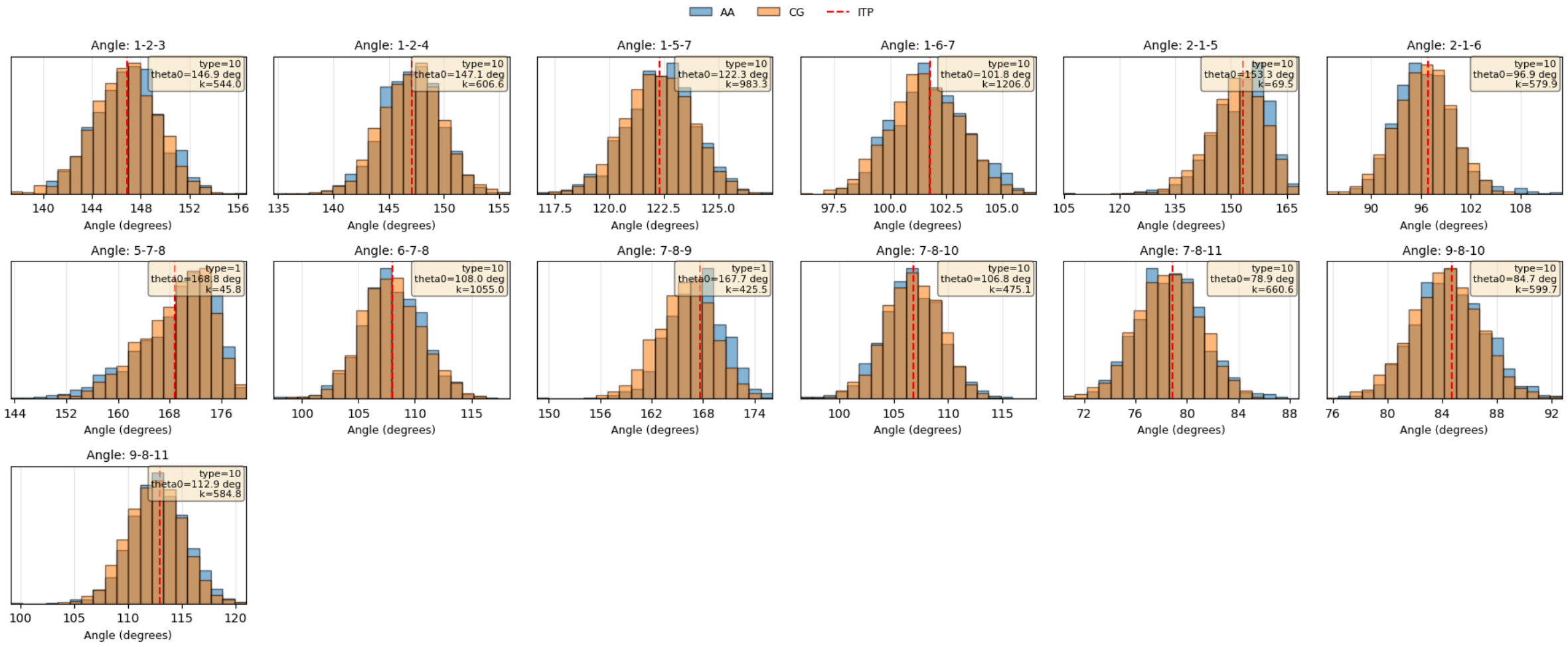
AA
CG
ITP
Angle: 1-2-3
Angle: 1-2-4
Angle: 1-5-7
Angle: 1-6-7
Angle: 2-1-5
Angle: 2-1-6
Angle: 5-7-8
Angle: 6-7-8
Angle: 7-8-9
Angle: 7-8-10
Angle: 7-8-11
Angle: 9-8-10
Angle: 9-8-11
Angle (degrees)

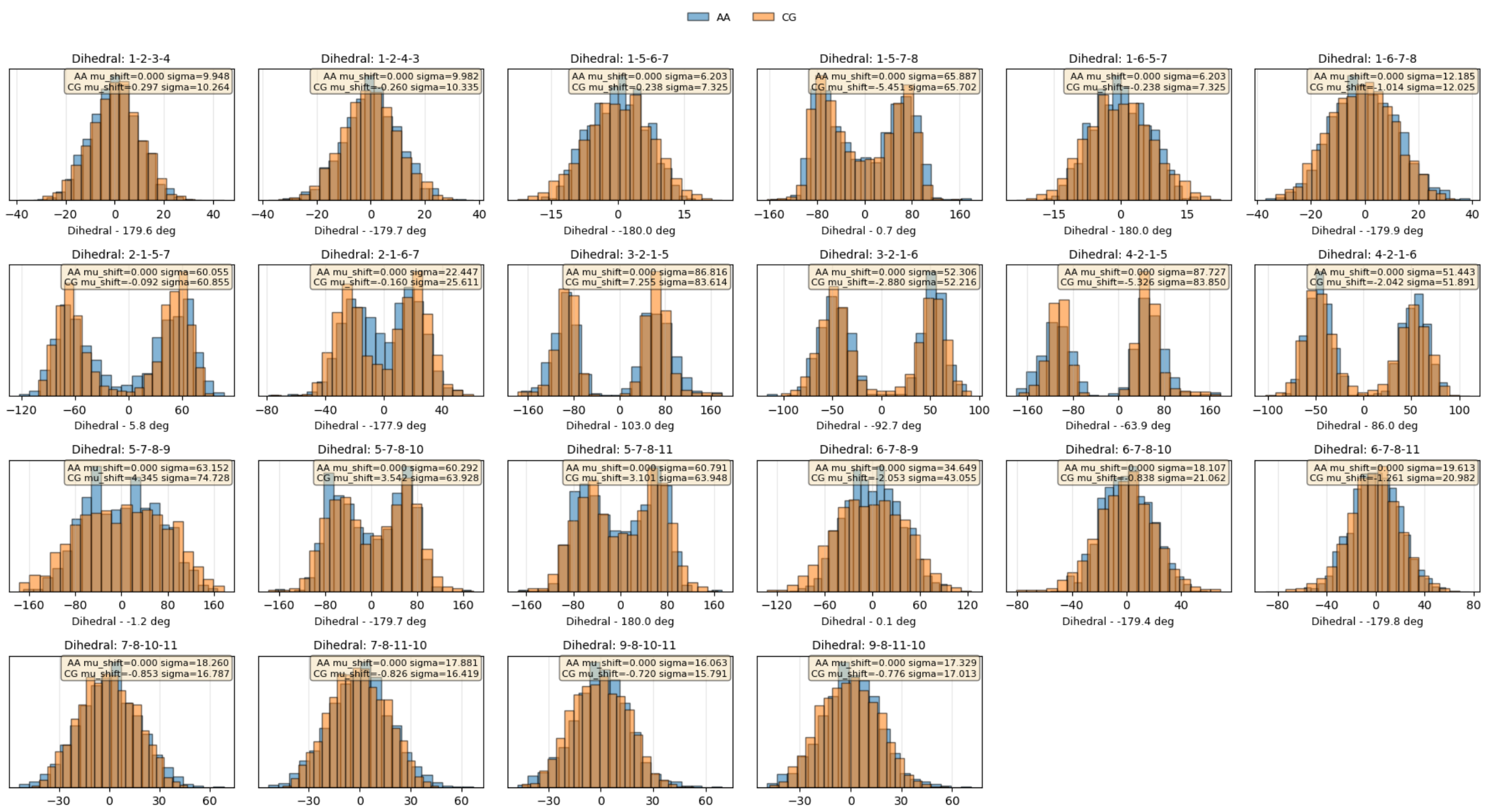
AA
CG
Dihedral: 1-2-3-4
Dihedral: 1-2-4-3
Dihedral: 1-5-6-7
Dihedral: 1-5-7-8
Dihedral: 1-6-5-7
Dihedral: 1-6-7-8
Dihedral: 2-1-5-7
Dihedral: 2-1-6-7
Dihedral: 3-2-1-5
Dihedral: 3-2-1-6
Dihedral: 4-2-1-5
Dihedral: 4-2-1-6
Dihedral: 5-7-8-9
Dihedral: 5-7-8-10
Dihedral: 5-7-8-11
Dihedral: 6-7-8-9
Dihedral: 6-7-8-10
Dihedral: 6-7-8-11
Dihedral: 7-8-10-11
Dihedral: 7-8-11-10
Dihedral: 9-8-10-11
Dihedral: 9-8-11-10

HEM

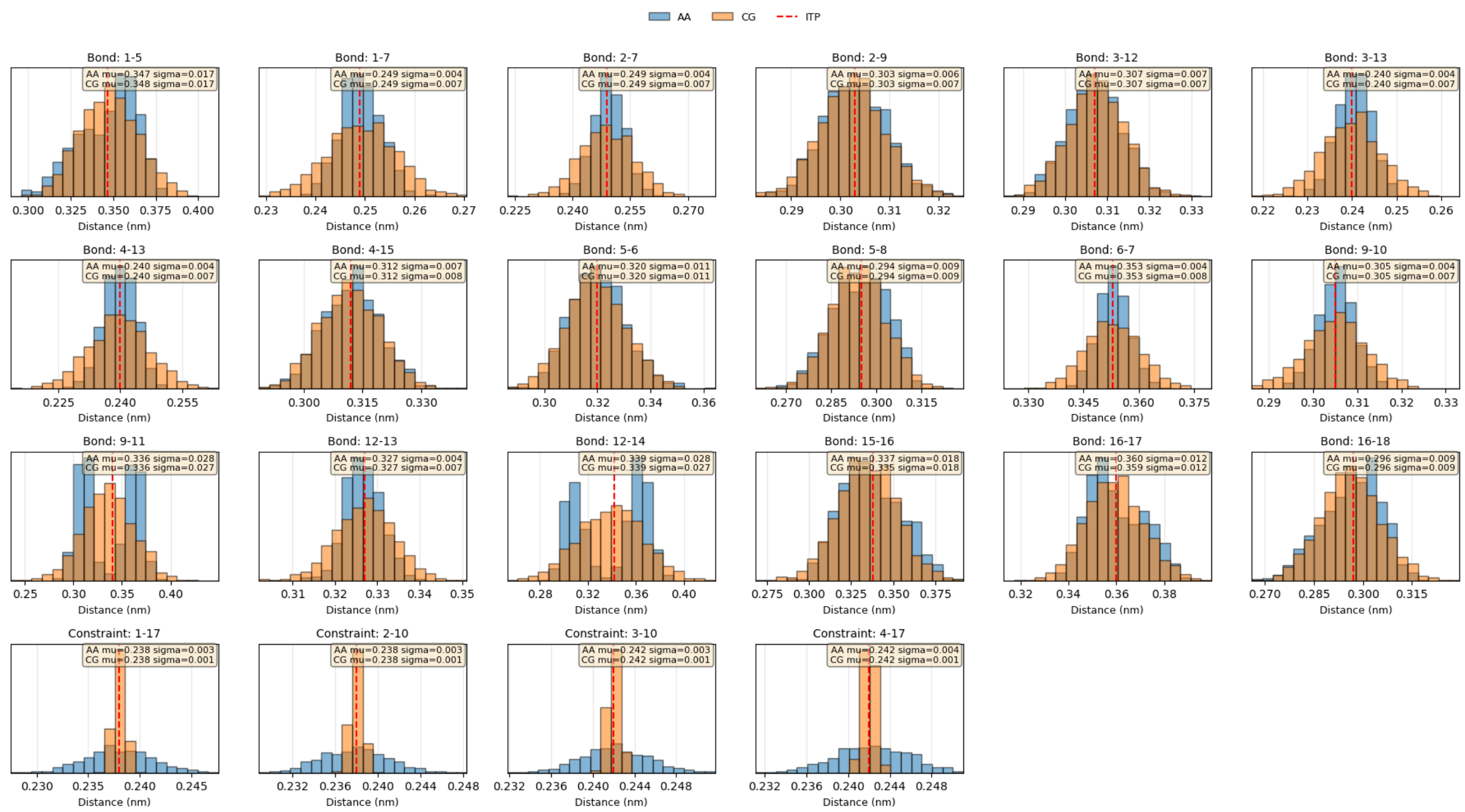

AA
CG
ITP
Bond: 1-5
AA mu=0.347 sigma=0.017
CG mu=0.348 sigma=0.017
Bond: 1-7
AA mu=0.249 sigma=0.004
CG mu=0.249 sigma=0.007
Bond: 2-7
AA mu=0.249 sigma=0.004
CG mu=0.249 sigma=0.007
Bond: 2-9
AA mu=0.303 sigma=0.006
CG mu=0.303 sigma=0.007
Bond: 3-12
AA mu=0.307 sigma=0.007
CG mu=0.307 sigma=0.007
Bond: 3-13
AA mu=0.240 sigma=0.004
CG mu=0.240 sigma=0.007
Bond: 4-13
AA mu=0.240 sigma=0.004
CG mu=0.240 sigma=0.007
Bond: 4-15
AA mu=0.312 sigma=0.007
CG mu=0.312 sigma=0.008
Bond: 5-6
AA mu=0.320 sigma=0.011
CG mu=0.320 sigma=0.011
Bond: 5-8
AA mu=0.294 sigma=0.009
CG mu=0.294 sigma=0.009
Bond: 6-7
AA mu=0.353 sigma=0.004
CG mu=0.353 sigma=0.008
Bond: 9-10
AA mu=0.305 sigma=0.004
CG mu=0.305 sigma=0.007
Bond: 9-11
AA mu=0.336 sigma=0.028
CG mu=0.336 sigma=0.027
Bond: 12-13
AA mu=0.327 sigma=0.004
CG mu=0.327 sigma=0.007
Bond: 12-14
AA mu=0.339 sigma=0.028
CG mu=0.339 sigma=0.027
Bond: 15-16
AA mu=0.337 sigma=0.018
CG mu=0.335 sigma=0.018
Bond: 16-17
AA mu=0.360 sigma=0.012
CG mu=0.359 sigma=0.012
Bond: 16-18
AA mu=0.296 sigma=0.009
CG mu=0.296 sigma=0.009
Constraint: 1-17
AA mu=0.238 sigma=0.003
CG mu=0.238 sigma=0.001
Constraint: 2-10
AA mu=0.238 sigma=0.003
CG mu=0.238 sigma=0.001
Constraint: 3-10
AA mu=0.242 sigma=0.003
CG mu=0.242 sigma=0.001
Constraint: 4-17
AA mu=0.242 sigma=0.004
CG mu=0.242 sigma=0.001
Distance (nm)

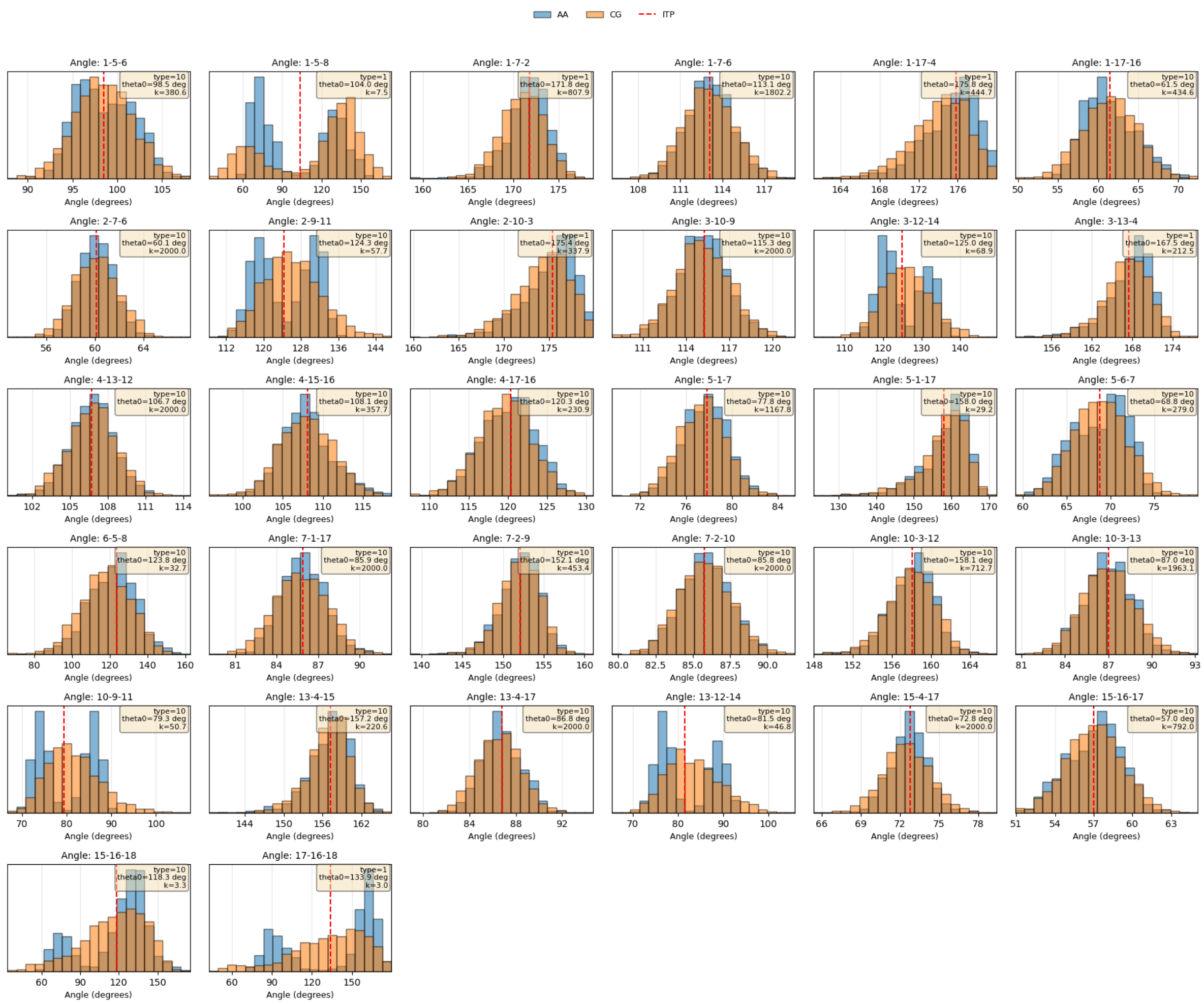
AA
CG
ITP
Angle: 1-5-6
type=10 theta0=98.5 deg k=380.6
Angle: 1-5-8
type=1 theta0=104.0 deg k=7.5
Angle: 1-7-2
type=1 theta0=171.8 deg k=807.9
Angle: 1-7-6
type=10 theta0=113.1 deg k=1802.2
Angle: 1-17-4
type=1 theta0=175.8 deg k=444.7
Angle: 1-17-16
type=10 theta0=61.5 deg k=434.6
Angle: 2-7-6
type=10 theta0=60.1 deg k=2000.0
Angle: 2-9-11
type=10 theta0=124.3 deg k=57.7
Angle: 2-10-3
type=1 theta0=175.4 deg k=337.9
Angle: 3-10-9
type=10 theta0=115.3 deg k=2000.0
Angle: 3-12-14
type=10 theta0=125.0 deg k=68.9
Angle: 3-13-4
type=1 theta0=167.5 deg k=212.5
Angle: 4-13-12
type=10 theta0=106.7 deg k=2000.0
Angle: 4-15-16
type=10 theta0=108.1 deg k=357.7
Angle: 4-17-16
type=10 theta0=120.3 deg k=230.9
Angle: 5-1-7
type=10 theta0=77.8 deg k=1167.8
Angle: 5-1-17
type=10 theta0=158.0 deg k=29.2
Angle: 5-6-7
type=10 theta0=68.8 deg k=279.0
Angle: 6-5-8
type=10 theta0=123.8 deg k=32.7
Angle: 7-1-17
type=10 theta0=85.9 deg k=2000.0
Angle: 7-2-9
type=10 theta0=152.1 deg k=453.4
Angle: 7-2-10
type=10 theta0=85.8 deg k=2000.0
Angle: 10-3-12
type=10 theta0=158.1 deg k=712.7
Angle: 10-3-13
type=10 theta0=87.0 deg k=1963.1
Angle: 10-9-11
type=10 theta0=79.3 deg k=50.7
Angle: 13-4-15
type=10 theta0=157.2 deg k=220.6
Angle: 13-4-17
type=10 theta0=86.8 deg k=2000.0
Angle: 13-12-14
type=10 theta0=81.5 deg k=46.8
Angle: 15-4-17
type=10 theta0=72.8 deg k=2000.0
Angle: 15-16-17
type=10 theta0=57.0 deg k=792.0
Angle: 15-16-18
type=10 theta0=118.3 deg k=3.3
Angle: 17-16-18
type=1 theta0=133.9 deg k=3.0
Angle (degrees)

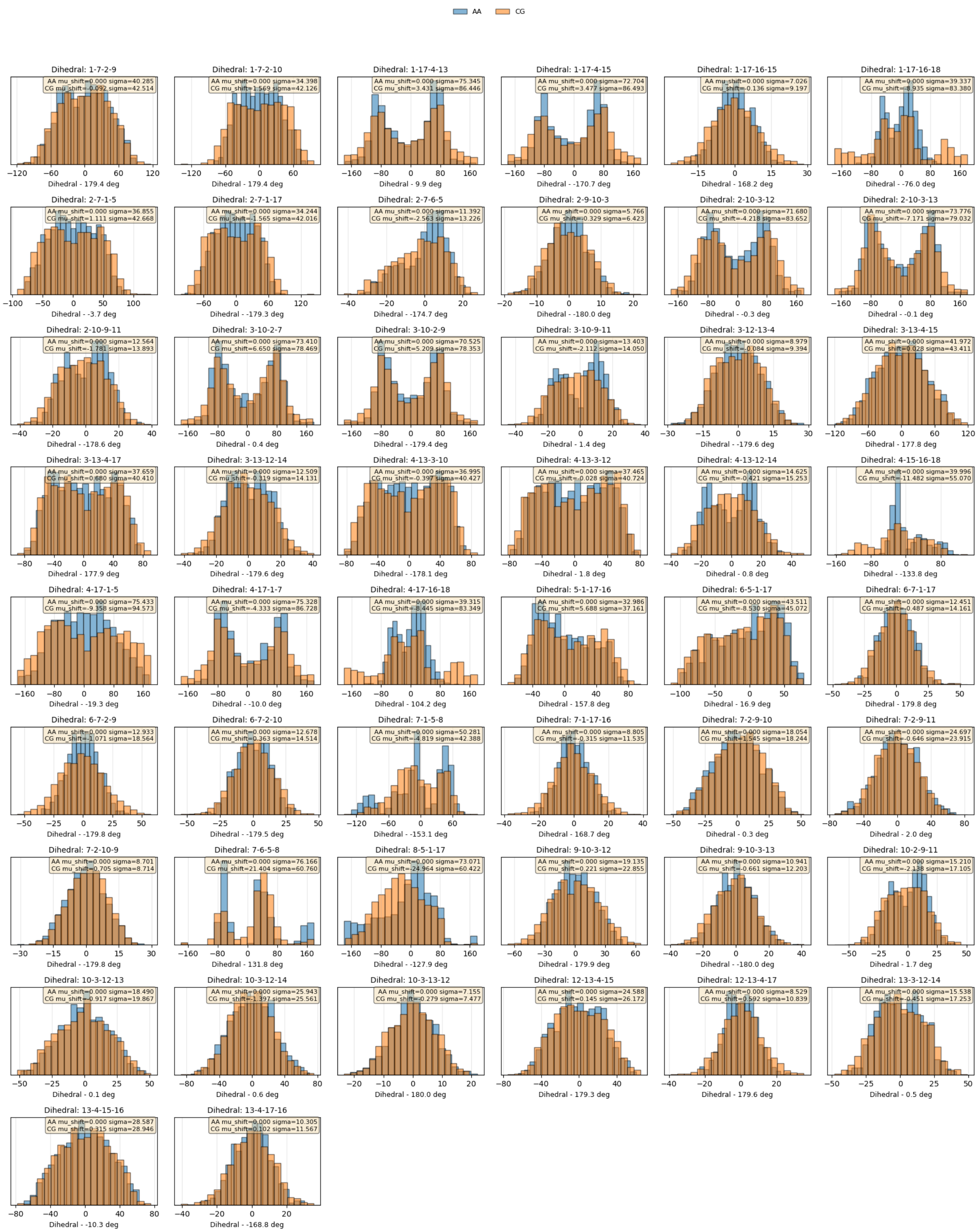

AA
CG
Dihedral: 1-7-2-9
Dihedral - 179.4 deg
Dihedral: 1-7-2-10
Dihedral - 179.4 deg
Dihedral: 1-17-4-13
Dihedral - 9.9 deg
Dihedral: 1-17-4-15
Dihedral - -170.7 deg
Dihedral: 1-17-16-15
Dihedral - 168.2 deg
Dihedral: 1-17-16-18
Dihedral - -76.0 deg
Dihedral: 2-7-1-5
Dihedral - -3.7 deg
Dihedral: 2-7-1-17
Dihedral - -179.3 deg
Dihedral: 2-7-6-5
Dihedral - -174.7 deg
Dihedral: 2-9-10-3
Dihedral - -180.0 deg
Dihedral: 2-10-3-12
Dihedral - -0.3 deg
Dihedral: 2-10-3-13
Dihedral - -0.1 deg
Dihedral: 2-10-9-11
Dihedral - -178.6 deg
Dihedral: 3-10-2-7
Dihedral - 0.4 deg
Dihedral: 3-10-2-9
Dihedral - -179.4 deg
Dihedral: 3-10-9-11
Dihedral - 1.4 deg
Dihedral: 3-12-13-4
Dihedral - -179.6 deg
Dihedral: 3-13-4-15
Dihedral - 177.8 deg
Dihedral: 3-13-4-17
Dihedral - 177.9 deg
Dihedral: 3-13-12-14
Dihedral - -179.6 deg
Dihedral: 4-13-3-10
Dihedral - -178.1 deg
Dihedral: 4-13-3-12
Dihedral - 1.8 deg
Dihedral: 4-13-12-14
Dihedral - 0.8 deg
Dihedral: 4-15-16-18
Dihedral - -133.8 deg
Dihedral: 4-17-1-5
Dihedral - -19.3 deg
Dihedral: 4-17-1-7
Dihedral - -10.0 deg
Dihedral: 4-17-16-18
Dihedral - 104.2 deg
Dihedral: 5-1-17-16
Dihedral - 157.8 deg
Dihedral: 6-5-1-17
Dihedral - 16.9 deg
Dihedral: 6-7-1-17
Dihedral - 179.8 deg
Dihedral: 6-7-2-9
Dihedral - -179.8 deg
Dihedral: 6-7-2-10
Dihedral - -179.5 deg
Dihedral: 7-1-5-8
Dihedral - -153.1 deg
Dihedral: 7-1-17-16
Dihedral - 168.7 deg
Dihedral: 7-2-9-10
Dihedral - 0.3 deg
Dihedral: 7-2-9-11
Dihedral - 2.0 deg
Dihedral: 7-2-10-9
Dihedral - -179.8 deg
Dihedral: 7-6-5-8
Dihedral - 131.8 deg
Dihedral: 8-5-1-17
Dihedral - -127.9 deg
Dihedral: 9-10-3-12
Dihedral - 179.9 deg
Dihedral: 9-10-3-13
Dihedral - -180.0 deg
Dihedral: 10-2-9-11
Dihedral - 1.7 deg
Dihedral: 10-3-12-13
Dihedral - 0.1 deg
Dihedral: 10-3-12-14
Dihedral - 0.6 deg
Dihedral: 10-3-13-12
Dihedral - 180.0 deg
Dihedral: 12-13-4-15
Dihedral - 179.3 deg
Dihedral: 12-13-4-17
Dihedral - 179.6 deg
Dihedral: 13-3-12-14
Dihedral - 0.5 deg
Dihedral: 13-4-15-16
Dihedral - -10.3 deg
Dihedral: 13-4-17-16
Dihedral - -168.8 deg

LIB

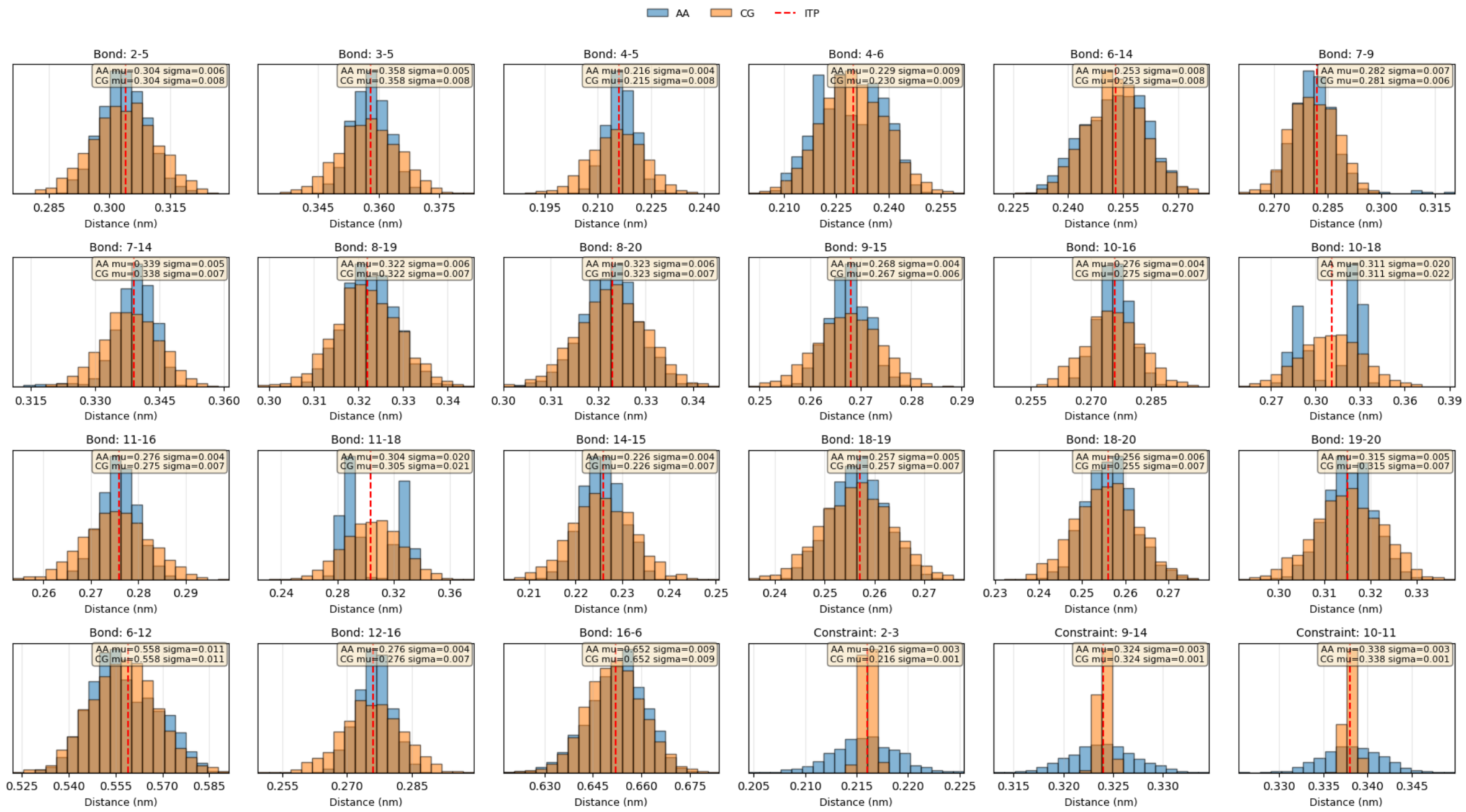
AA
CG
ITP
Bond: 2-5
AA mu=0.304 sigma=0.006
CG mu=0.304 sigma=0.008
Bond: 3-5
AA mu=0.358 sigma=0.005
CG mu=0.358 sigma=0.008
Bond: 4-5
AA mu=0.216 sigma=0.004
CG mu=0.215 sigma=0.008
Bond: 4-6
AA mu=0.229 sigma=0.009
CG mu=0.230 sigma=0.009
Bond: 6-14
AA mu=0.253 sigma=0.008
CG mu=0.253 sigma=0.008
Bond: 7-9
AA mu=0.282 sigma=0.007
CG mu=0.281 sigma=0.006
Bond: 7-14
AA mu=0.339 sigma=0.005
CG mu=0.338 sigma=0.007
Bond: 8-19
AA mu=0.322 sigma=0.006
CG mu=0.322 sigma=0.007
Bond: 8-20
AA mu=0.323 sigma=0.006
CG mu=0.323 sigma=0.007
Bond: 9-15
AA mu=0.268 sigma=0.004
CG mu=0.267 sigma=0.006
Bond: 10-16
AA mu=0.276 sigma=0.004
CG mu=0.275 sigma=0.007
Bond: 10-18
AA mu=0.311 sigma=0.020
CG mu=0.311 sigma=0.022
Bond: 11-16
AA mu=0.276 sigma=0.004
CG mu=0.275 sigma=0.007
Bond: 11-18
AA mu=0.304 sigma=0.020
CG mu=0.305 sigma=0.021
Bond: 14-15
AA mu=0.226 sigma=0.004
CG mu=0.226 sigma=0.007
Bond: 18-19
AA mu=0.257 sigma=0.005
CG mu=0.257 sigma=0.007
Bond: 18-20
AA mu=0.256 sigma=0.006
CG mu=0.255 sigma=0.007
Bond: 19-20
AA mu=0.315 sigma=0.005
CG mu=0.315 sigma=0.007
Bond: 6-12
AA mu=0.558 sigma=0.011
CG mu=0.558 sigma=0.011
Bond: 12-16
AA mu=0.276 sigma=0.004
CG mu=0.276 sigma=0.007
Bond: 16-6
AA mu=0.652 sigma=0.009
CG mu=0.652 sigma=0.009
Constraint: 2-3
AA mu=0.216 sigma=0.003
CG mu=0.216 sigma=0.001
Constraint: 9-14
AA mu=0.324 sigma=0.003
CG mu=0.324 sigma=0.001
Constraint: 10-11
AA mu=0.338 sigma=0.003
CG mu=0.338 sigma=0.001
Distance (nm)

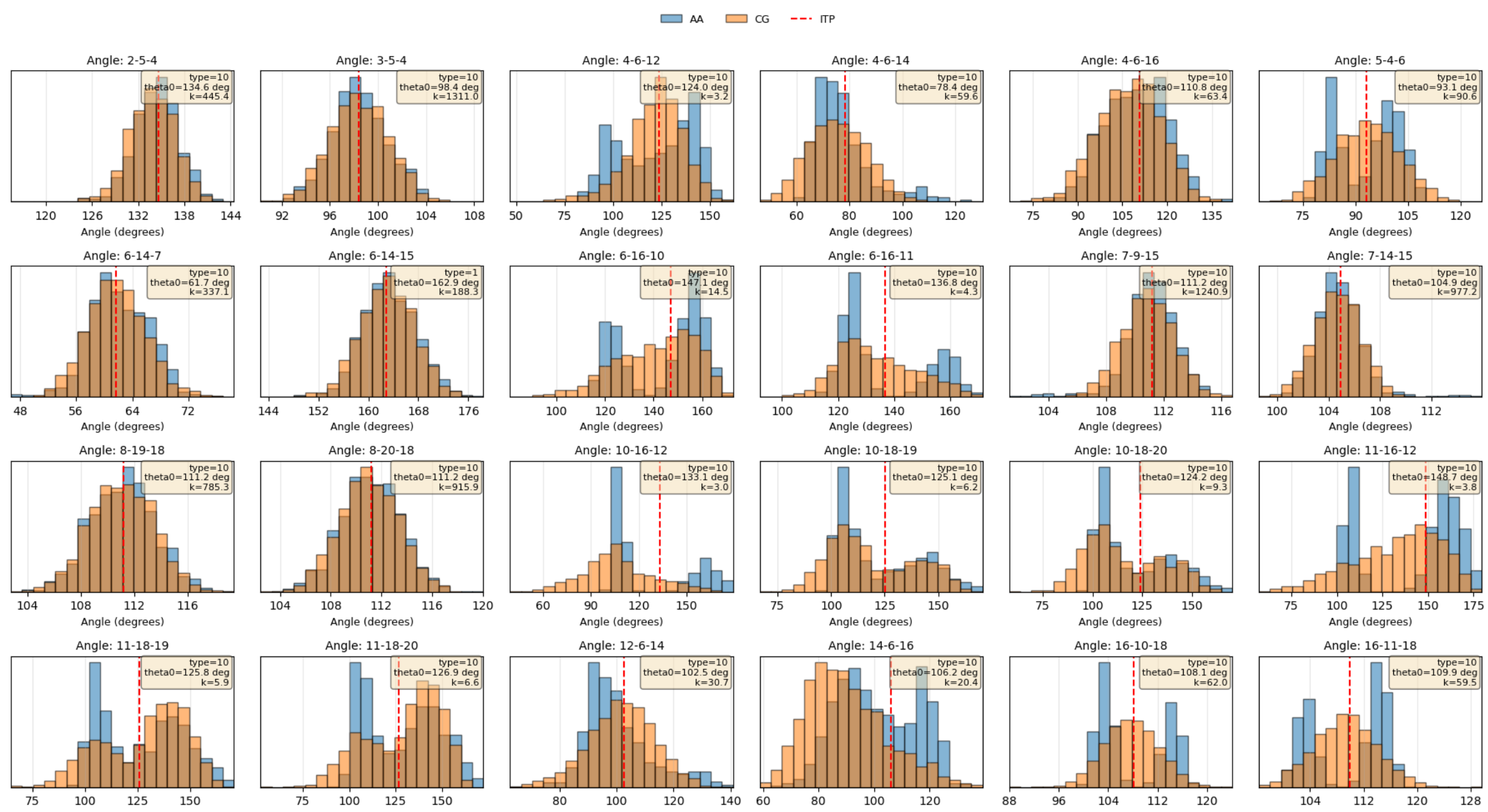
AA
CG
ITP
Angle: 2-5-4
type=10 theta0=134.6 deg k=445.4
Angle: 3-5-4
type=10 theta0=98.4 deg k=1311.0
Angle: 4-6-12
type=10 theta0=124.0 deg k=3.2
Angle: 4-6-14
type=10 theta0=78.4 deg k=59.6
Angle: 4-6-16
type=10 theta0=110.8 deg k=63.4
Angle: 5-4-6
type=10 theta0=93.1 deg k=90.6
Angle: 6-14-7
type=10 theta0=61.7 deg k=337.1
Angle: 6-14-15
type=1 theta0=162.9 deg k=188.3
Angle: 6-16-10
type=10 theta0=147.1 deg k=14.5
Angle: 6-16-11
type=10 theta0=136.8 deg k=4.3
Angle: 7-9-15
type=10 theta0=111.2 deg k=1240.9
Angle: 7-14-15
type=10 theta0=104.9 deg k=977.2
Angle: 8-19-18
type=10 theta0=111.2 deg k=785.3
Angle: 8-20-18
type=10 theta0=111.2 deg k=915.9
Angle: 10-16-12
type=10 theta0=133.1 deg k=3.0
Angle: 10-18-19
type=10 theta0=125.1 deg k=6.2
Angle: 10-18-20
type=10 theta0=124.2 deg k=9.3
Angle: 11-16-12
type=10 theta0=148.7 deg k=3.8
Angle: 11-18-19
type=10 theta0=125.8 deg k=5.9
Angle: 11-18-20
type=10 theta0=126.9 deg k=6.6
Angle: 12-6-14
type=10 theta0=102.5 deg k=30.7
Angle: 14-6-16
type=10 theta0=106.2 deg k=20.4
Angle: 16-10-18
type=10 theta0=108.1 deg k=62.0
Angle: 16-11-18
type=10 theta0=109.9 deg k=59.5
Angle (degrees)

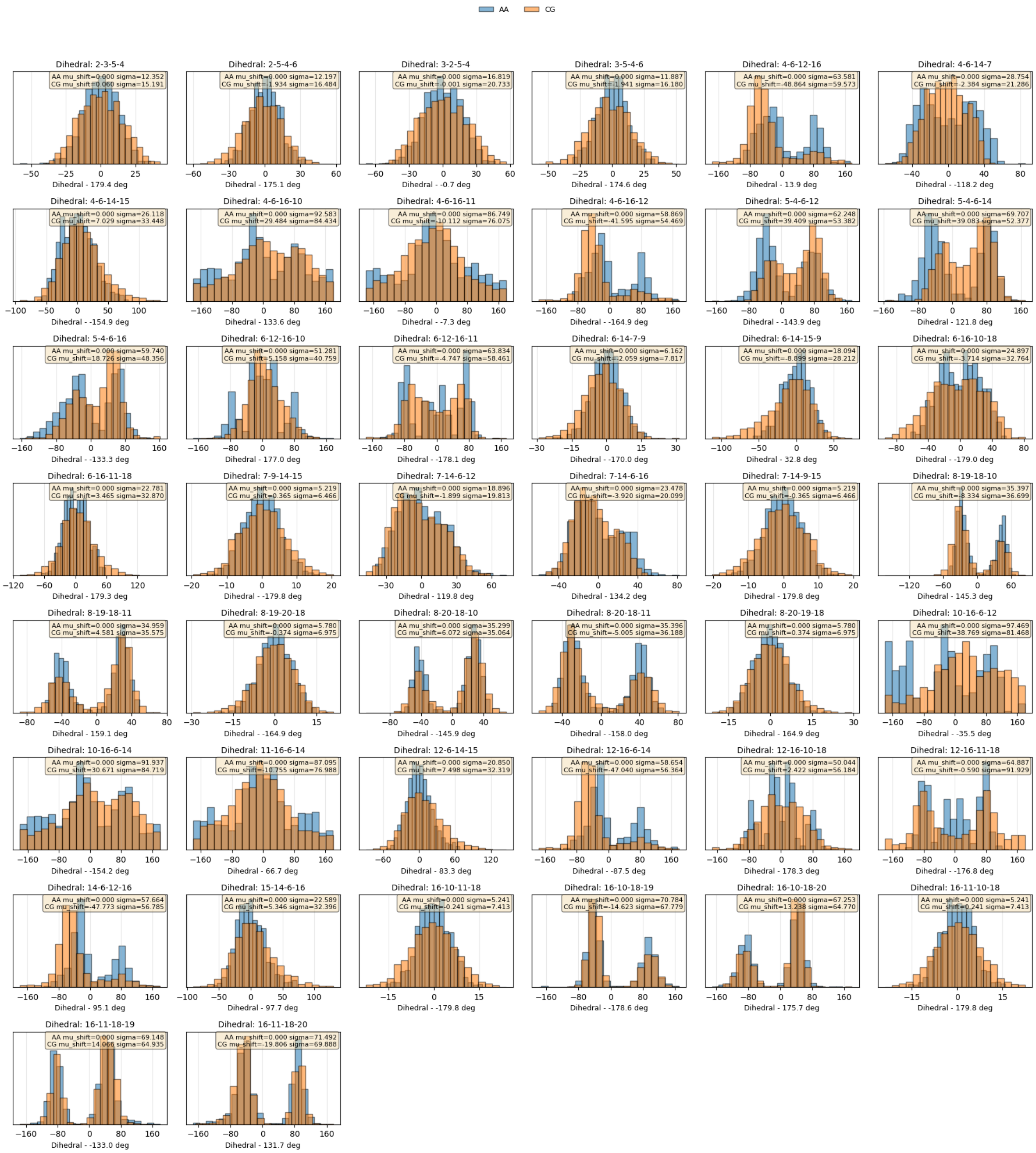
AA
CG
Dihedral: 2-3-5-4
AA mu_shift=0.000 sigma=12.352
CG mu_shift=0.060 sigma=15.191
Dihedral - 179.4 deg
Dihedral: 2-5-4-6
AA mu_shift=0.000 sigma=12.197
CG mu_shift=-1.934 sigma=16.484
Dihedral - 175.1 deg
Dihedral: 3-2-5-4
AA mu_shift=0.000 sigma=16.819
CG mu_shift=-0.001 sigma=20.733
Dihedral - -0.7 deg
Dihedral: 3-5-4-6
AA mu_shift=0.000 sigma=11.887
CG mu_shift=-1.941 sigma=16.180
Dihedral - 174.6 deg
Dihedral: 4-6-12-16
AA mu_shift=0.000 sigma=63.581
CG mu_shift=-48.864 sigma=59.573
Dihedral - 13.9 deg
Dihedral: 4-6-14-7
AA mu_shift=0.000 sigma=28.754
CG mu_shift=-2.384 sigma=21.286
Dihedral - -118.2 deg
Dihedral: 4-6-14-15
AA mu_shift=0.000 sigma=26.118
CG mu_shift=7.029 sigma=33.448
Dihedral - -154.9 deg
Dihedral: 4-6-16-10
AA mu_shift=0.000 sigma=92.583
CG mu_shift=29.484 sigma=84.434
Dihedral - 133.6 deg
Dihedral: 4-6-16-11
AA mu_shift=0.000 sigma=86.749
CG mu_shift=-10.112 sigma=76.075
Dihedral - -7.3 deg
Dihedral: 4-6-16-12
AA mu_shift=0.000 sigma=58.869
CG mu_shift=-41.595 sigma=54.469
Dihedral - -164.9 deg
Dihedral: 5-4-6-12
AA mu_shift=0.000 sigma=62.248
CG mu_shift=39.409 sigma=53.382
Dihedral - -143.9 deg
Dihedral: 5-4-6-14
AA mu_shift=0.000 sigma=69.707
CG mu_shift=39.083 sigma=52.377
Dihedral - 121.8 deg
Dihedral: 5-4-6-16
AA mu_shift=0.000 sigma=59.740
CG mu_shift=18.726 sigma=48.356
Dihedral - -133.3 deg
Dihedral: 6-12-16-10
AA mu_shift=0.000 sigma=51.281
CG mu_shift=5.158 sigma=40.759
Dihedral - 177.0 deg
Dihedral: 6-12-16-11
AA mu_shift=0.000 sigma=63.834
CG mu_shift=-4.747 sigma=58.461
Dihedral - -178.1 deg
Dihedral: 6-14-7-9
AA mu_shift=0.000 sigma=6.162
CG mu_shift=-2.059 sigma=7.817
Dihedral - -170.0 deg
Dihedral: 6-14-15-9
AA mu_shift=0.000 sigma=18.094
CG mu_shift=-8.899 sigma=28.212
Dihedral - 32.8 deg
Dihedral: 6-16-10-18
AA mu_shift=0.000 sigma=24.897
CG mu_shift=-3.714 sigma=32.764
Dihedral - -179.0 deg
Dihedral: 6-16-11-18
AA mu_shift=0.000 sigma=22.781
CG mu_shift=3.465 sigma=32.870
Dihedral - 179.3 deg
Dihedral: 7-9-14-15
AA mu_shift=0.000 sigma=5.219
CG mu_shift=0.365 sigma=6.466
Dihedral - -179.8 deg
Dihedral: 7-14-6-12
AA mu_shift=0.000 sigma=18.896
CG mu_shift=-1.899 sigma=19.813
Dihedral - 119.8 deg
Dihedral: 7-14-6-16
AA mu_shift=0.000 sigma=23.478
CG mu_shift=-3.920 sigma=20.099
Dihedral - 134.2 deg
Dihedral: 7-14-9-15
AA mu_shift=0.000 sigma=5.219
CG mu_shift=-0.365 sigma=6.466
Dihedral - 179.8 deg
Dihedral: 8-19-18-10
AA mu_shift=0.000 sigma=35.397
CG mu_shift=-8.334 sigma=36.699
Dihedral - 145.3 deg
Dihedral: 8-19-18-11
AA mu_shift=0.000 sigma=34.959
CG mu_shift=4.581 sigma=35.575
Dihedral - 159.1 deg
Dihedral: 8-19-20-18
AA mu_shift=0.000 sigma=5.780
CG mu_shift=-0.374 sigma=6.975
Dihedral - -164.9 deg
Dihedral: 8-20-18-10
AA mu_shift=0.000 sigma=35.299
CG mu_shift=6.072 sigma=35.064
Dihedral - -145.9 deg
Dihedral: 8-20-18-11
AA mu_shift=0.000 sigma=35.396
CG mu_shift=-5.005 sigma=36.188
Dihedral - -158.0 deg
Dihedral: 8-20-19-18
AA mu_shift=0.000 sigma=5.780
CG mu_shift=0.374 sigma=6.975
Dihedral - 164.9 deg
Dihedral: 10-16-6-12
AA mu_shift=0.000 sigma=97.469
CG mu_shift=38.769 sigma=81.468
Dihedral - -35.5 deg
Dihedral: 10-16-6-14
AA mu_shift=0.000 sigma=91.937
CG mu_shift=30.671 sigma=84.719
Dihedral - -154.2 deg
Dihedral: 11-16-6-14
AA mu_shift=0.000 sigma=87.095
CG mu_shift=-10.755 sigma=76.988
Dihedral - 66.7 deg
Dihedral: 12-6-14-15
AA mu_shift=0.000 sigma=20.850
CG mu_shift=7.498 sigma=32.319
Dihedral - 83.3 deg
Dihedral: 12-16-6-14
AA mu_shift=0.000 sigma=58.654
CG mu_shift=-47.040 sigma=56.364
Dihedral - -87.5 deg
Dihedral: 12-16-10-18
AA mu_shift=0.000 sigma=50.044
CG mu_shift=2.422 sigma=56.184
Dihedral - 178.3 deg
Dihedral: 12-16-11-18
AA mu_shift=0.000 sigma=64.887
CG mu_shift=-0.590 sigma=91.929
Dihedral - -176.8 deg
Dihedral: 14-6-12-16
AA mu_shift=0.000 sigma=57.664
CG mu_shift=-47.773 sigma=56.785
Dihedral - 95.1 deg
Dihedral: 15-14-6-16
AA mu_shift=0.000 sigma=22.589
CG mu_shift=5.346 sigma=32.396
Dihedral - 97.7 deg
Dihedral: 16-10-11-18
AA mu_shift=0.000 sigma=5.241
CG mu_shift=-0.241 sigma=7.413
Dihedral - -179.8 deg
Dihedral: 16-10-18-19
AA mu_shift=0.000 sigma=70.784
CG mu_shift=-14.623 sigma=67.779
Dihedral - -178.6 deg
Dihedral: 16-10-18-20
AA mu_shift=0.000 sigma=67.253
CG mu_shift=13.238 sigma=64.770
Dihedral - 175.7 deg
Dihedral: 16-11-10-18
AA mu_shift=0.000 sigma=5.241
CG mu_shift=0.241 sigma=7.413
Dihedral - 179.8 deg
Dihedral: 16-11-18-19
AA mu_shift=0.000 sigma=69.148
CG mu_shift=14.066 sigma=64.935
Dihedral - -133.0 deg
Dihedral: 16-11-18-20
AA mu_shift=0.000 sigma=71.492
CG mu_shift=-19.806 sigma=69.888
Dihedral - 131.7 deg

THC

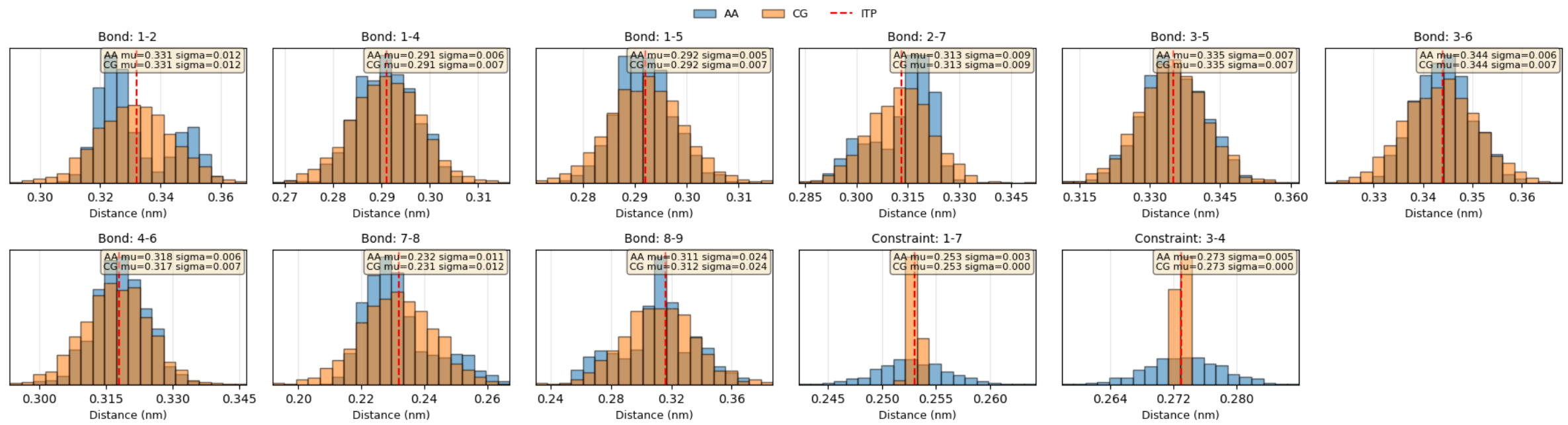
AA
CG
ITP
Bond: 1-2
AA mu=0.331 sigma=0.012
CG mu=0.331 sigma=0.012
Bond: 1-4
AA mu=0.291 sigma=0.006
CG mu=0.291 sigma=0.007
Bond: 1-5
AA mu=0.292 sigma=0.005
CG mu=0.292 sigma=0.007
Bond: 2-7
AA mu=0.313 sigma=0.009
CG mu=0.313 sigma=0.009
Bond: 3-5
AA mu=0.335 sigma=0.007
CG mu=0.335 sigma=0.007
Bond: 3-6
AA mu=0.344 sigma=0.006
CG mu=0.344 sigma=0.007
Bond: 4-6
AA mu=0.318 sigma=0.006
CG mu=0.317 sigma=0.007
Bond: 7-8
AA mu=0.232 sigma=0.011
CG mu=0.231 sigma=0.012
Bond: 8-9
AA mu=0.311 sigma=0.024
CG mu=0.312 sigma=0.024
Constraint: 1-7
AA mu=0.253 sigma=0.003
CG mu=0.253 sigma=0.000
Constraint: 3-4
AA mu=0.273 sigma=0.005
CG mu=0.273 sigma=0.000
Distance (nm)

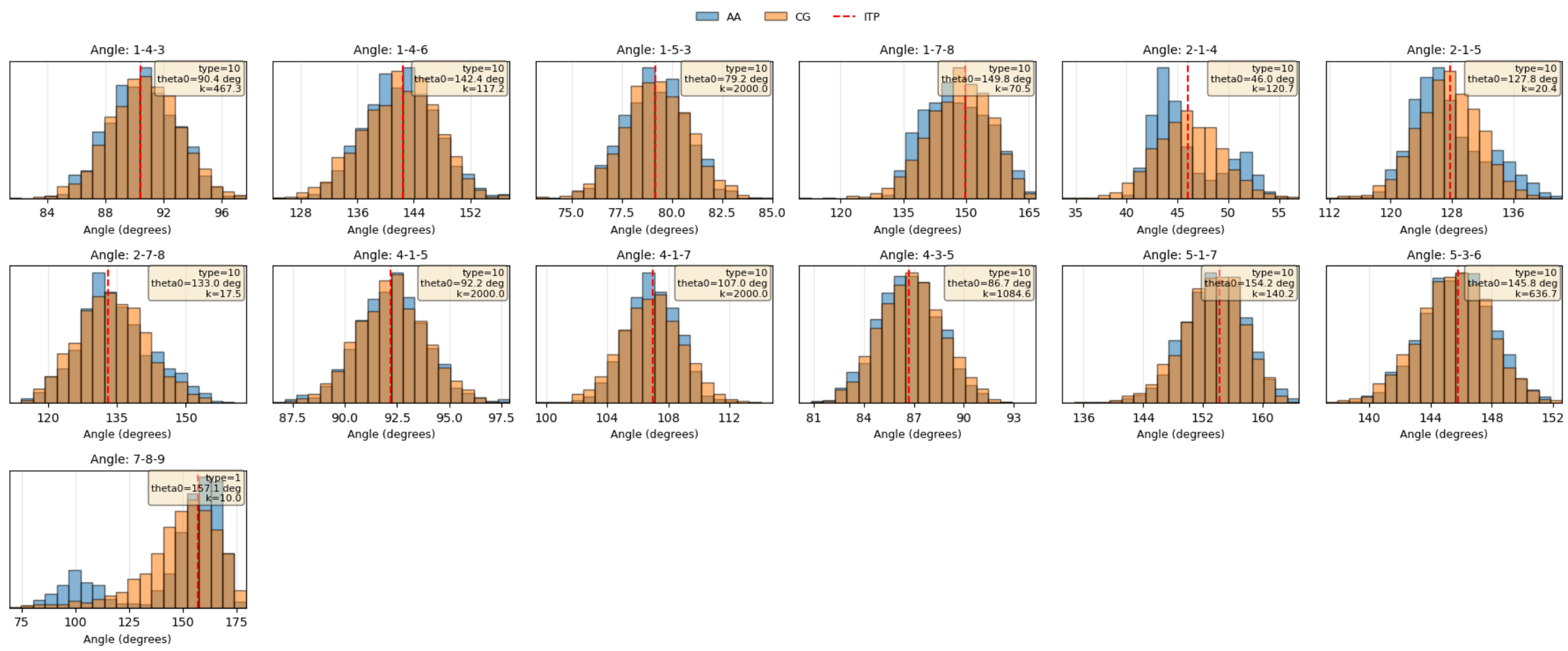
AA
CG
ITP
Angle: 1-4-3
type=10
theta0=90.4 deg
k=467.3
Angle: 1-4-6
type=10
theta0=142.4 deg
k=117.2
Angle: 1-5-3
type=10
theta0=79.2 deg
k=2000.0
Angle: 1-7-8
type=10
theta0=149.8 deg
k=70.5
Angle: 2-1-4
type=10
theta0=46.0 deg
k=120.7
Angle: 2-1-5
type=10
theta0=127.8 deg
k=20.4
Angle: 2-7-8
type=10
theta0=133.0 deg
k=17.5
Angle: 4-1-5
type=10
theta0=92.2 deg
k=2000.0
Angle: 4-1-7
type=10
theta0=107.0 deg
k=2000.0
Angle: 4-3-5
type=10
theta0=86.7 deg
k=1084.6
Angle: 5-1-7
type=10
theta0=154.2 deg
k=140.2
Angle: 5-3-6
type=10
theta0=145.8 deg
k=636.7
Angle: 7-8-9
type=1
theta0=157.1 deg
k=10.0
Angle (degrees)

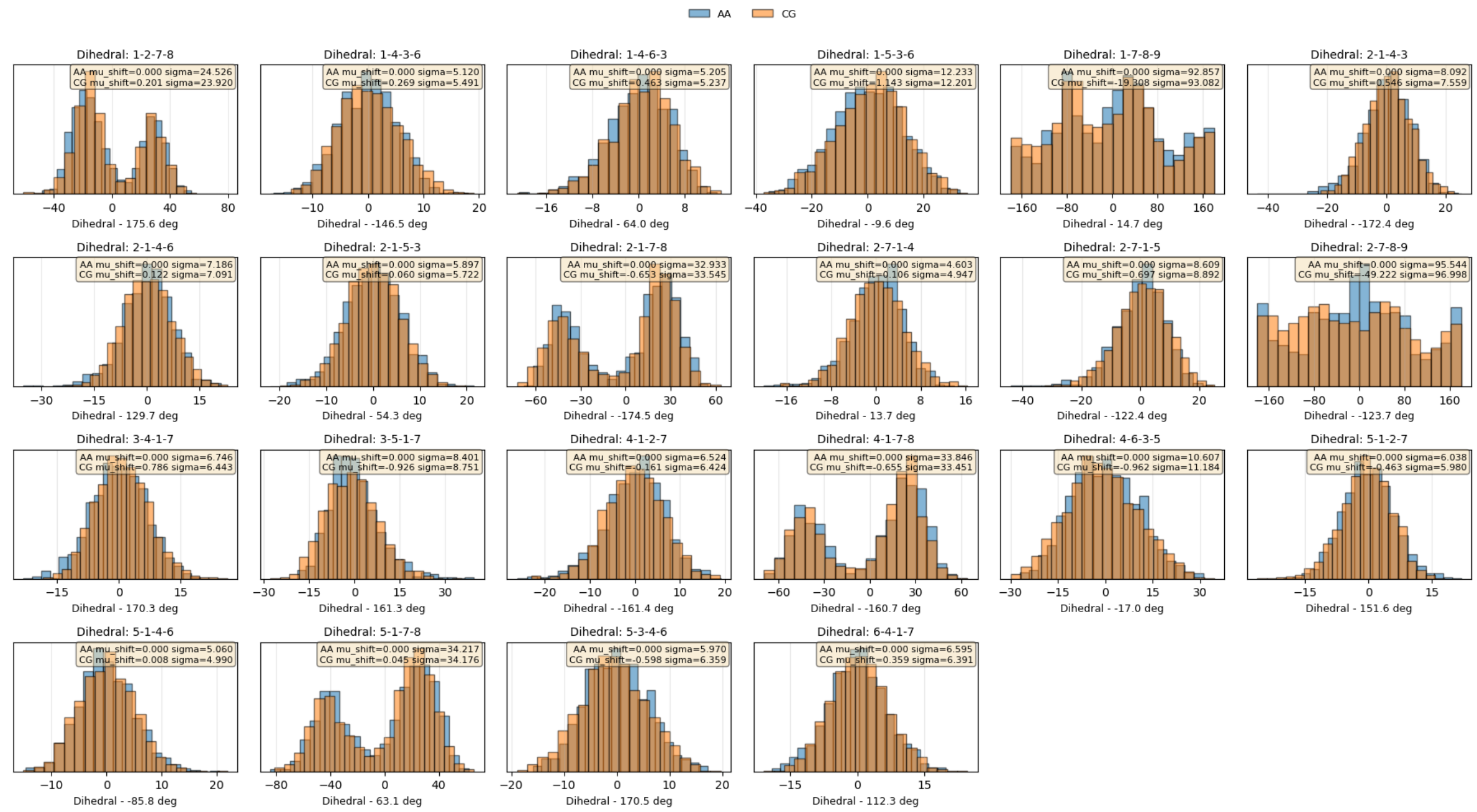

References

Best, Robert B., Gerhard Hummer, and William A. Eaton. 2013. "Native Contacts Determine Protein Folding Mechanisms in Atomistic Simulations." *Proceedings of the National Academy of Sciences* 110 (44): 17874–79. https://doi.org/10.1073/pnas.1311599110.

Gowers, Richard J., Max Linke, Jonathan Barnoud, et al. 2016. "MDAnalysis: A Python Package for the Rapid Analysis of Molecular Dynamics Simulations." *Proceedings of the 15th Python in Science Conference*, 98–105. https://doi.org/10.25080/Majora-629e541a-00e.

Harris, Charles R., K. Jarrod Millman, Stéfan J. van der Walt, et al. 2020. "Array Programming with NumPy." *Nature* 585: 357–62. https://doi.org/10.1038/s41586-020-2649-2.

McGibbon, Robert T., Kyle A. Beauchamp, Matthew P. Harrigan, et al. 2015. "MDTraj: A Modern Open Library for the Analysis of Molecular Dynamics Trajectories." *Biophysical Journal* 109 (8): 1528–32. https://doi.org/10.1016/j.bpj.2015.08.015.

Shrake, A., and J. A. Rupley. 1973. "Environment and Exposure to Solvent of Protein Atoms. Lysozyme and Insulin." *Journal of Molecular Biology* 79 (2): 351–71. https://doi.org/10.1016/0022-2836(73)90011-9.
